# 2D nanosheets of layered double perovskites: synthesis, photostable bright orange emission and photoluminescence blinking†

**DOI:** 10.1039/d3sc02506c

**Published:** 2023-06-20

**Authors:** Aditya Bhardwaj, Kaushik Kundu, Ranjan Sasmal, Paribesh Acharyya, Jayita Pradhan, Simanta Kalita, Sarit S. Agasti, Kanishka Biswas

**Affiliations:** a New Chemistry Unit and School of Advanced Materials Jawaharlal Nehru Centre for Advanced Scientific Research (JNCASR) Jakkur P.O. Bangalore 560064 India sagasti@jncasr.ac.in kanishka@jncasr.ac.in; b Chemistry and Physics of Materials Unit, JNCASR Jakkur P.O. Bangalore 560064 India

## Abstract

Lead (Pb)-free layered double perovskites (LDPs) with exciting optical properties and environmental stability have sparked attention in optoelectronics, but their high photoluminescence (PL) quantum yield and understanding of the PL blinking phenomenon at the single particle level are still elusive. Herein, we not only demonstrate a hot-injection route for the synthesis of two-dimensional (2D) ∼2–3 layer thick nanosheets (NSs) of LDP, Cs_4_CdBi_2_Cl_12_ (pristine), and its partially Mn-substituted analogue [*i.e.*, Cs_4_Cd_0.6_Mn_0.4_Bi_2_Cl_12_ (Mn-substituted)], but also present a solvent-free mechanochemical synthesis of these samples as bulk powders. Bright and intense orange emission has been perceived for partially Mn-substituted 2D NSs with a relatively high PL quantum yield (PLQY) of ∼21%. The PL and lifetime measurements both at cryogenic (77 K) and room temperatures were employed to understand the de-excitation pathways of charge carriers. With the implementation of super-resolved fluorescence microscopy and time-resolved single particle tracking, we identified the occurrence of metastable non-radiative recombination channels in a single NS. In contrast to the rapid photo-bleaching that resulted in a PL blinking-like nature of the controlled pristine NS, the 2D NS of the Mn-substituted sample displayed negligible photo-bleaching with suppression of PL fluctuation under continuous illumination. The blinking-like nature in pristine NSs appeared due to a dynamic equilibrium flanked by the active and in-active states of metastable non-radiative channels. However, the partial substitution of Mn^2+^ stabilized the in-active state of the non-radiative channels, which increased the PLQY and suppressed PL fluctuation and photo-bleaching events in Mn-substituted NSs.

## Abstract 2

Few-layer thick 2D nanosheets of Cs_4_Cd_0.6_Mn_0.4_Bi_2_Cl_12_ are synthesized *via* solution-based colloidal method. It displays intense orange emission and distinct suppression of photoluminescence fluctuation with negligible photo-bleaching.

## Graphical abstract

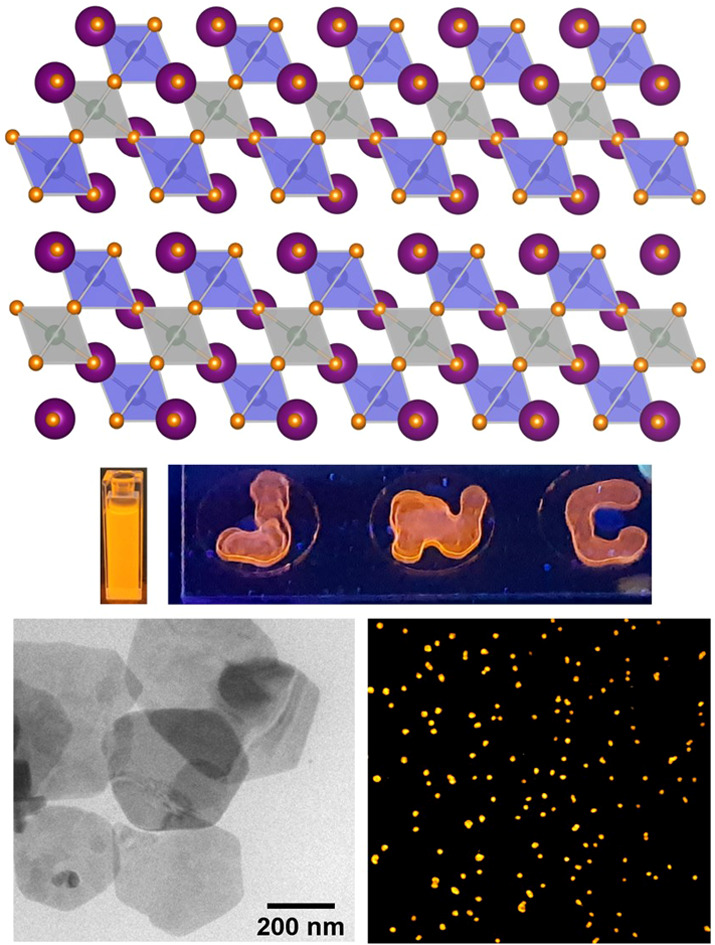

## Introduction

Metal halide perovskite nanostructures have received significant attention owing to their outstanding optoelectronic properties.^1^ In spite of their unique properties, the foremost shortcoming is the presence of lead (Pb), which obstructs them from further applications.^2^ Hence, the search for environmentally-friendly replacements that maintain the remarkable optical properties of Pb-based perovskite halides has become an increasingly significant research area. Promising approaches included isovalent substitution of Pb^2+^ by less- or non-toxic metal ions, like Sn^2+^ and Ge^2+^.^3^ However, facile oxidation of these cations under ambient conditions leads to the inferior stability of crystal structures and impedes their applications. Subsequently, strategies for employing cations with an oxidation state other than +2 in place of Pb^2+^ in perovskites are being extensively explored. The lower dimensional A_3_M_2_^III^X_9_-type perovskites,^4^ and paired monovalent-trivalent cation based double perovskites, A_2_M^I^M^III^X_6_ [where M(i) and M(iii) correspond to Ag^+^, Cu^+^, Na^+^, *etc.* and Bi^3+^, Sb^3+^, In^3+^, *etc.*, respectively],^5^ are being actively studied, and have shown promising properties.

Recently, dimensional reduction and heterovalent substitution resulted in layered double perovskite (LDP) halides, which can be reckoned as the two-dimensional (2D) variety of double perovskites or the double-metal version of layered perovskites.^6^ Different from the conventional double perovskites, LDPs integrate the divalent and trivalent cations into 2D structures, which further led to the chemical and structural diversity. Additionally, the dimensional reduction can engender modifications in the electronic structure.^6^ Solis-Ibarra and co-workers initially synthesized a mixed-metal 〈111〉-oriented layered double perovskite with the general formula of A_*n*+1_[B(ii)B′(iii)]_*n*_X_3*n*+3_, where *n* > 2 (*i.e.*, Cs_4_CuSb_2_Cl_12_).^7^ Subsequently, few LDPs are available, which are either computationally proposed or experimentally synthesized.^6*a*,8^ Till now, the bulk form of LDPs (*e.g.*, A_4_M^II^M^III^_2_X_12_) has been synthesized mainly by acid precipitation methods.^6*b*^ Despite the great promise of this family of materials, very few reports have explored the synthesis of LDP nanostructures by the solution-based colloidal method.^9^ Recently, colloidal synthesis of Cs_4_M(ii)Bi_2_Cl_12_ [M(ii) = Cd, Mn] nanocrystals (NCs) was reported using the hot-injection method; however, a maximum photoluminescence quantum yield (PLQY) of only 4.6% was achieved for the Cs_4_(Cd_1−*x*_Mn_*x*_)Bi_2_Cl_12_ NCs.^9*b*^ Therefore, facile synthesis strategies for both the bulk form and the nanostructures (*e.g.*, 2D nanoplates or nanosheets) of such LDPs should be developed to improve the PLQY and to understand the PL properties at the single particle level.

Herein, we report a simple, solvent-free, scalable, and environment friendly all-solid-state mechanochemical synthesis of the bulk powders of LDPs, Cs_4_CdBi_2_Cl_12_ (pristine) and a partially Mn-substituted analogue [Cs_4_Cd_0.6_Mn_0.4_Bi_2_Cl_12_]. More importantly, we have synthesized the 2–3 layer thick 2D nanosheets (NSs) of the pristine and Mn-substituted analogue by the one-pot solution-based hot-injection method using benzoyl chloride as a halide precursor. These materials showed high thermal and environmental stability. The 2D NSs of the Mn-substituted analogue showed a relatively high PLQY of ∼21% at room temperature. A deeper understanding of the charge-carrier dynamics in these 2D NSs was achieved through time-resolved PL measurements both at room and cryogenic (77 K) temperatures. Super-resolved fluorescence microscopy and time-resolved single particle tracking were employed to investigate the PL blinking event in 2D NSs of the pristine and Mn-substituted analogue at the single particle (herein, NS) level, which is a key process to control the emission efficiency. Photo-bleaching and temporal intermittency (blinking) behaviours have been perceived in controlled pristine NSs, whereas the single NS of the Mn-substituted analogue displayed relatively suppressed PL fluctuation characteristics. An enriched PL intensity with a high photon count was evidenced in Mn-substituted NSs *via* structured illumination microscopy (SIM) accompanied by negligible photo-bleaching behaviour under continuous light illumination.

## Results and discussion

Here, we first discuss the solvent-free mechanochemical synthesis, stability, and optical properties of bulk polycrystalline powders, and subsequently, we present the colloidal synthesis of 2D nanosheets (NSs) of Cs_4_CdBi_2_Cl_12_ (pristine), and Cs_4_Cd_0.6_Mn_0.4_Bi_2_Cl_12_ (Mn-substituted analogue); and super-resolved fluorescence microscopy results to understand the PL fluctuation behaviour at the single NS level.

**Scheme 1:**
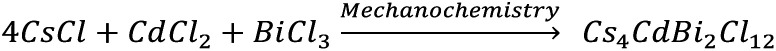


**Scheme 2:**
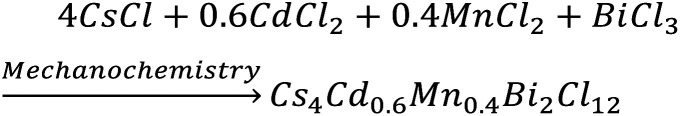


To synthesize the bulk powders of the pristine and Mn-substituted analogue, we have used CsCl, CdCl_2_, MnCl_2_, and BiCl_3_ as precursors in the appropriate stoichiometric ratio. Typically, a stoichiometric amount of the metal chloride precursors was mixed and ground mechanically in a mortar and pestle for 2 hours in an ambient laboratory environment (Schemes 1 and 2). Previously, such bulk powders have been synthesized by the precipitation method using concentrated hydrochloric acid.^8*a*,10^ In contrast, our all-solid state mechanochemical approach is solvent-free, and scalable to ∼1 g of pure crystalline product.

**Fig. 1:**
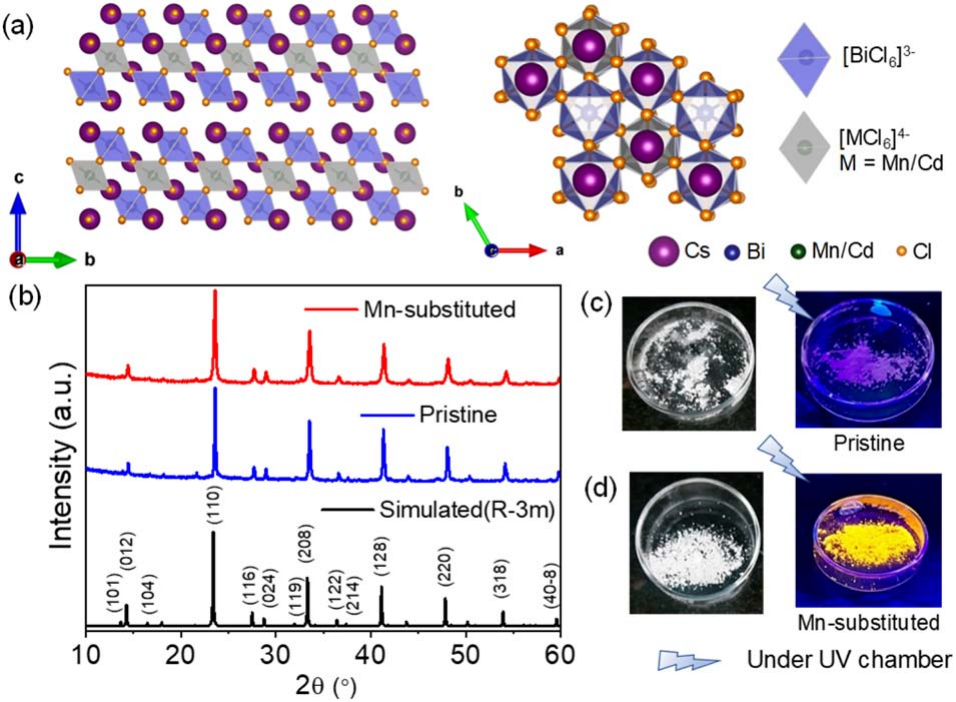
(a) Crystal structure of layered double perovskite, Cs_4_M(ii)Bi_2_Cl_12_, where M = Cd/Mn. (b) Powder XRD pattern of mechanochemically synthesized Cs_4_CdBi_2_Cl_12_ (pristine) and Cs_4_Cd_0.6_Mn_0.4_Bi_2_Cl_12_ (Mn-substituted) bulk powders. Images (c) and (d) are the physical appearances of pristine and Mn-substituted samples, respectively, under day light and UV light.

The powder X-ray diffraction (PXRD) pattern in Fig. 1b for the mechanochemically synthesized bulk powders was perfectly matched with the rhombohedral phase of pristine (space group *R*3̄*m*), which indicates the phase purity of the obtained product. A right shift of XRD peaks in 2*θ* was evidenced for the bulk Mn-substituted analogue, which corresponds to the smaller ionic radius of Mn^2+^ (83 pm) as compared to that of Cd^2+^ (95 pm). Cs_4_Cd_1−*x*_Mn_*x*_Bi_2_Cl_12_ (where, *x* = 0 and 0.4) exhibits a layered double perovskite (LDP) crystal structure, which can be derived from the conventional cubic ABX_3_ perovskite structure. Cs_4_Cd_1−*x*_Mn_*x*_Bi_2_Cl_12_ possesses a unique structure with a layer of divalent metal halide octahedra (*i.e.*, [M(ii)X_6_]^4−^, M(ii) = Cd, and/or Mn), sandwiched between the two layers of trivalent metal halide octahedra (*i.e.*, [M(iii)X_6_]^3−^, M(iii) = Bi). The overall tri-layer motifs are separated by top and bottom vacancy (V) layers [an alternating M(iii)–M(ii)–M(iii)–V pattern] along the [001] crystal direction (the original [111] direction of the cubic lattice) of the LDP, as shown in Fig. 1a.^6*a*,11^

The visual appearances of as-synthesized bulk powders are demonstrated in Fig. 1c and d. The Mn-substituted powders showed intense orange emission, whereas pristine powders displayed low intense purple colour under UV light. To investigate the environmental stability, the PXRD patterns of bulk pristine and Mn-substituted powders were monitored after 30 days of exposure to the ambient conditions (Fig. S1a and b, ESI†), which confirmed the absence of degradation. The thermal stabilities of these mechanochemically synthesized bulk powders were investigated by thermogravimetric analysis (TGA), as shown in Fig. S2a (ESI†). Both the samples showed stability up to 400 °C, after which approximately 19% and 23% weight losses occurred at ∼450 °C for pristine and Mn-substituted analogue, respectively, and further decomposition has been observed thereafter. The characteristic Raman active vibrational modes arising from the different metal octahedra in pristine and Mn-substituted samples were ascertained from the implementation of Raman spectroscopy (Fig. S2b, ESI†). For the pristine sample, the Raman active vibrational bands at 118, 268, and 291 cm^−1^ appeared due to the breathing (T_2g_), asymmetric stretching (E_g_) and symmetric stretching (A_1g_) modes of BiCl_6_ octahedra, respectively,^12^ whereas the bands at 147 and 243 cm^−1^ appeared from the Cd–Cl bending and stretching modes, respectively.^13^ The shoulder observed in the 118 and 147 cm^−1^ bands of the doped samples is probably due to the partial Mn^2+^ substitution. In addition, a small hump at 224 cm^−1^ is observed in the Raman spectra of the Mn-substituted sample, which might have appeared from the symmetric stretching of the Mn–Cl bond.^14^ Moreover, the X-ray photoelectron spectroscopy (XPS) measurement confirmed the expected oxidation state of all elements (Cs, Cd, Mn, Bi and Cl) in the Mn-substituted sample (Fig. S3a–e, ESI†).

**Fig. 2:**
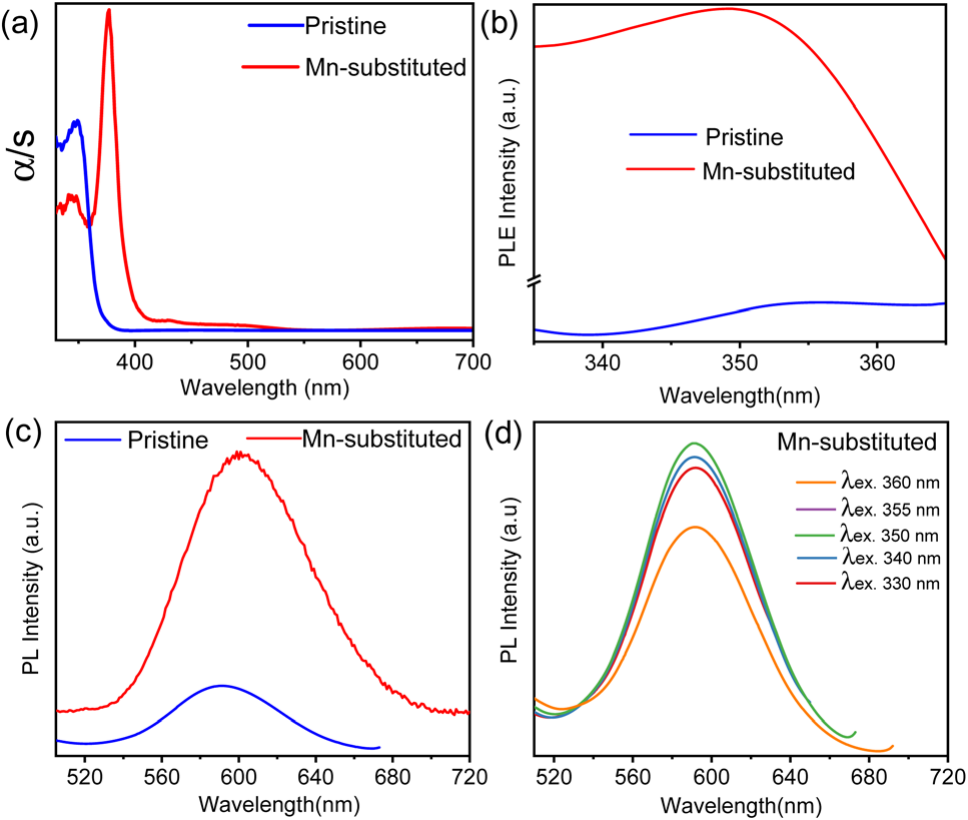
(a) Solid-state UV/vis absorption spectra, (b) solid-state PL excitation (PLE) plot at the emission maximum, and (c) solid-state room-temperature PL emission plot for Cs_4_CdBi_2_Cl_12_ (pristine) and Cs_4_Cd_0.6_Mn_0.4_Bi_2_Cl_12_ (Mn-substituted) bulk powders at an excitation wavelength of 350 nm. (d) Solid-state PL emission spectra for Mn-substituted bulk powders at different excitation wavelengths.

The optical properties were investigated for both pristine and Mn-substituted bulk powder samples by diffuse reflectance and PL spectroscopic techniques in the solid state. Electronic absorption spectra of pristine and Mn-substituted samples are presented in Fig. 2a. The optical band gaps of both samples were estimated from the onset of the absorption edge by extrapolating the linear region, which appeared to be ∼372 nm (∼3.33 eV) nm and ∼408 nm (∼3.08 eV) for pristine and Mn-substituted analogue, respectively.^8*a*^ The absorption spectrum of the Mn-substituted sample involves two extra weaker peaks in the visible region at ∼490 and ∼430 nm, whereas these peaks are absent in the pristine sample (Fig. S4, ESI†). The additional peaks in the Mn-substituted sample can be assigned to the spin-forbidden ^6^A_1_(S) → ^4^T_1_(G) and ^6^A_1_(S) → ^4^T_2_(G) d–d transitions in the high spin Mn^2+^ ion, respectively. The solid-state room-temperature PL spectra of the bulk pristine sample displayed a PL band centred at 601 nm (Fig. 2c), while the bulk Mn-substituted sample showed highly intense broad PL centred at 595 nm with an emission line-width of 60 nm (Fig. 2c). The representative orange PL emission of the Mn-substituted sample is ascribed to the spin and parity forbidden ^4^T_1g_ (G) → ^6^A_1g_ (S) transition within the 3d shell of the octahedrally coordinated Mn^2+^.^10^ Moreover, the emission peak maxima in PL spectra of the Mn-substituted sample were found to be independent of the excitation wavelength as shown in Fig. 2d, which designates a single radiative decay pathway.^8*a*,10^ Furthermore, the PL excitation (PLE) spectrum of both samples monitored at *λ*^max^_em_ matched well with the absorption spectrum (Fig. 2b).^8*a*^ However, the broadband and highly Stokes shifted PL emission of the Mn-substituted sample could be due to the energy transfer from the intrinsic dark self-trap excitonic (STE) state to the Mn^2+^ luminescent centre.^15^

**Fig. 3:**
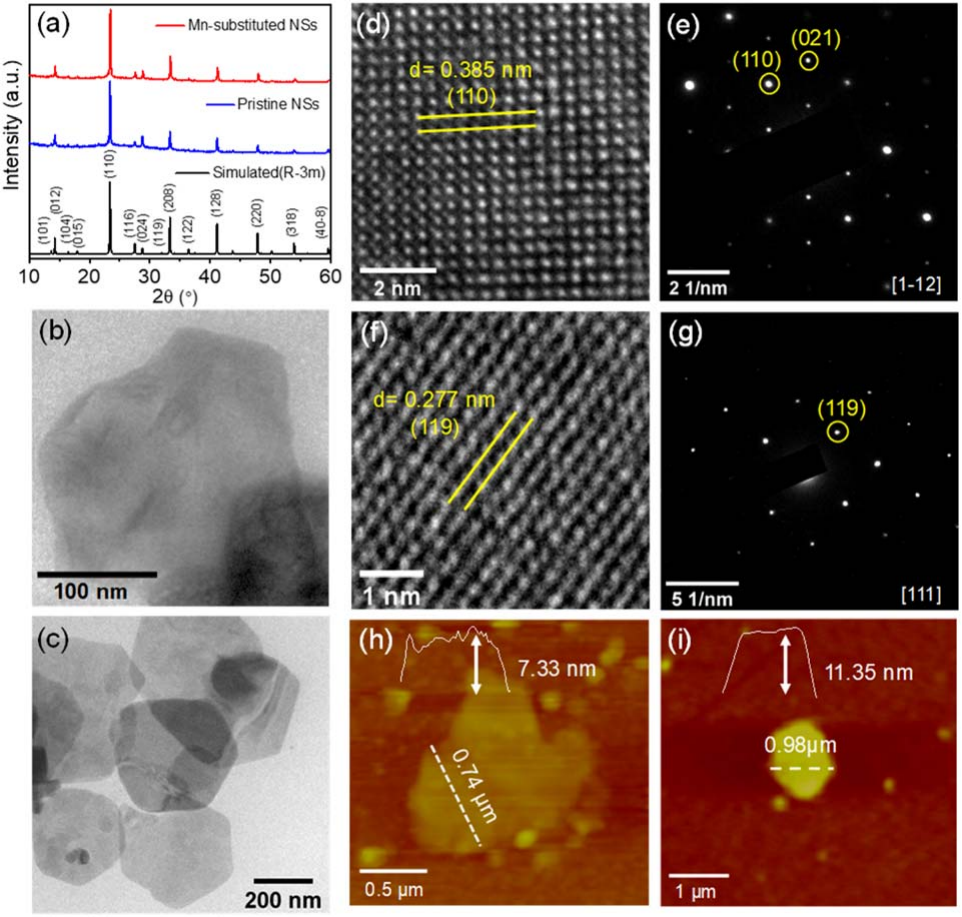
(a) PXRD pattern of Cs_4_CdBi_2_Cl_12_ (pristine) and Cs_4_Cd_0.6_Mn_0.4_Bi_2_Cl_12_ (Mn-substituted) nanostructured samples. (b) and (c) TEM images of pristine and Mn-substituted nanosheet (NS) samples, respectively. (d) and (f) depict the HRTEM images, and (e) and (g) represent the SAED patterns of the respective NS samples. (h) and (i) represent AFM images of pristine and Mn-substituted NS samples, respectively.

We have employed the solution-based hot-injection method for the colloidal synthesis of pristine and Mn-substituted nanostructured samples. In a typical synthesis of Cs_4_Cd_*x*_Mn_1−*x*_Bi_2_Cl_12_, an appropriate proportion of Cs_2_CO_3_, Cd(OAc)_2_·2H_2_O and/or Mn(OAc)_2_ and Bi(OAc)_3_ was dissolved in 1-octadecene (ODE), following which oleic acid (OA) and oleylamine (OAm) with a ratio of 2 : 1 were added and the reaction was kept at 120 °C under N_2_ for 1 hour. Subsequently, the temperature was increased to 135 °C and benzoyl chloride was injected. The reaction was then quenched in an ice water bath and the as-synthesized product was washed several times with isopropanol. The detailed synthesis strategy is presented in Scheme S1 (ESI†). The PXRD patterns of the as-synthesized nanostructures in Fig. 3a are well matched with the simulated pattern, which confirms the phase-purity. The shift of the PXRD peak to a slightly higher 2*θ* angle for the Mn-substituted sample compared to that of the pristine sample is due to lattice contraction caused by incorporation of Mn^2+^ at the Cd^2+^ site of the pristine sample. The actual composition of the Mn-substituted sample is further estimated from the ICP-AES analysis, which closely matched with the nominal stoichiometry (Table S1, ESI†). The detailed structural analysis was carried out using the composition obtained from ICP-AES analysis (*i.e.*, Cs_4_Cd_0.67_Mn_0.33_Bi_2_Cl_12_), and the pristine sample by employing Rietveld refinement [Fig. S5 and Tables S2–S3 (ESI†)]. The PXRD pattern of both the nanostructured samples after 30 days of exposure to ambient conditions suggests decent environmental stability (Fig. S6, ESI†). The final products were dispersed in toluene and checked under ambient light and UV light illumination. The toluene-dispersed Mn-substituted nanostructured sample exhibited strong orange emission as compared to the low-intense purple emission of the pristine sample under UV light, as presented in Fig. S7a (ESI†).

To obtain information on the presence of added ligands (OA and OAm) on the surface of nanostructures, Fourier transform infrared spectroscopy (FTIR) was employed. Both samples showed similar FTIR spectra, as shown in Fig. S7b (ESI†). The FTIR spectra confirmed the characteristic modes of the oleyl group [CH_3_(CH_2_)_7_–CH=CH–(CH_2_)_8−_], which exists in both OA and OAm. The peaks at ∼2854 and 2920 cm^−1^ correspond to the symmetric and asymmetric stretching modes in –CH_2_, respectively. The *υ*_c=c_ stretching mode is also evident as a less intense peak at 1635 cm^−1^. As expected, the 1710 cm^−1^ peak appeared from the *υ*_c=o_ of the carboxylic group of OA. The 3175 cm^−1^ and 1517 cm^−1^ modes arose from the stretching and scissoring of NH_2_ groups of OAm, respectively. Moreover, the distinct 1460 cm^−1^ and 1378 cm^−1^ peaks appeared from the C–H bending vibration of the hydrocarbon chain of OA/OAm.^1*g*^ Therefore, the FTIR study confirms the existence of the ligands on the surface of nanostructured samples.

Transmission electron microscopy (TEM) revealed the 2D nanosheet (NS) morphology for both pristine and Mn-substituted samples (see Fig. 3b and c, respectively). High-resolution TEM (HRTEM) analysis revealed the *d*-spacing values from lattice fringes to be 0.385 nm and 0.277 nm (Fig. 3d and f), which correspond to the (110) and (119) crystal planes of pristine and Mn-substituted NSs, respectively. The selected area electron diffraction (SAED) patterns of the pristine and Mn-substituted NSs further confirm the single crystalline nature, with the diffraction spots corresponding to the [1–12] and [111] zone axis of the rhombohedral phase, respectively (Fig. 3e and g). Additionally, the atomic force microscopy (AFM) analysis was executed to investigate the thickness of NSs (Fig. 3h and i), in which the height profiles were found to be 7.33 and 11.35 nm in pristine and Mn-substituted NSs, respectively. These observations suggested that the NSs are two to three-layers thick (thickness of one layer corresponds to 3.35 nm). The lateral dimension of the NS ranges from 300 to 700 nm. The field-emission scanning electron microscopy (FESEM) images and EDAX elemental mapping for the two samples further confirmed the uniform distribution of all elements (Cs, Cd and/or Mn, Bi, and Cl) in both samples (Fig. S8 and S9, ESI†). Subsequently, the Mn-substituted NS sample was characterized by the electron paramagnetic resonance (EPR) measurement, as illustrated in Fig. S10 (ESI†). The single broad EPR resonance peak without any hyperfine splitting might be ascribed to the stronger magnetic interactions.^10^ The obtained *g* value of ∼1.99 suggests the presence of Mn^2+^ (electronic configuration: [Ar]3d^5^, *g* ∼ 2.0) in the corresponding NS sample.^16^

**Fig. 4:**
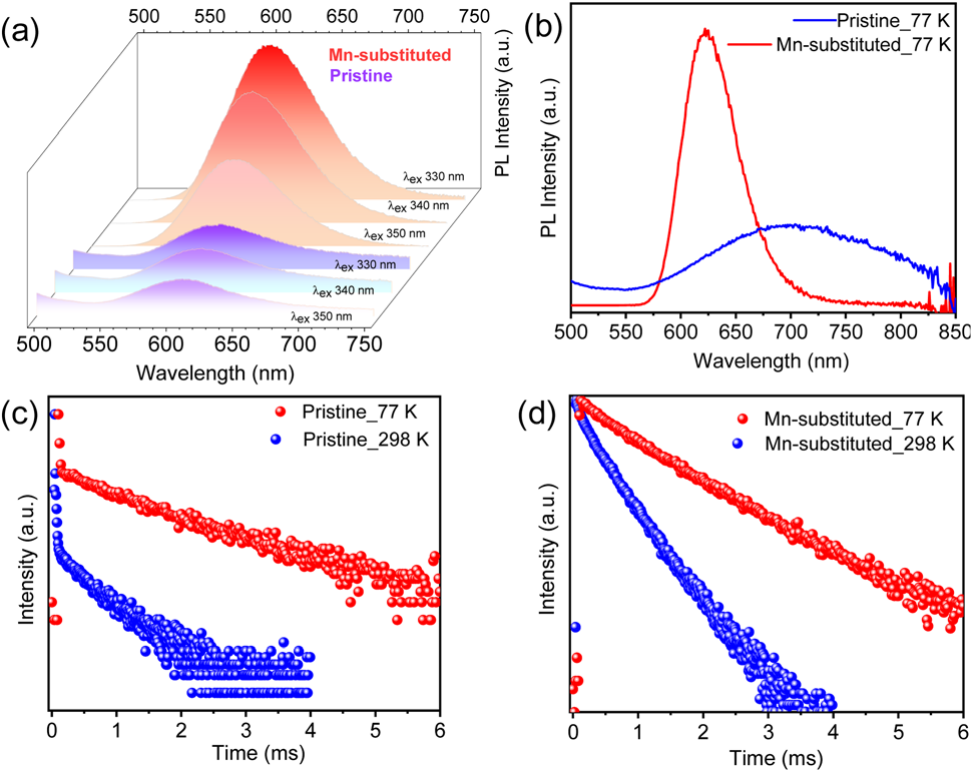
(a) Room temperature PL spectra of Cs_4_CdBi_2_Cl_12_ (pristine) and Cs_4_Cd_0.6_Mn_0.4_Bi_2_Cl_12_ (Mn-substituted) nanosheet (NS) samples at different excitation wavelengths. (b) PL spectra of pristine and Mn-substituted NS samples at 77 K. (c) and (d) Comparative time-resolved PL decay curves at 298 K and 77 K for pristine and Mn-substituted NS samples, respectively.

The optical properties of pristine and Mn-substituted NS samples were examined in the solution phase (dispersed in toluene). The UV-vis absorption spectra in Fig. S11a (ESI†) display a distinct and sharp absorption band maximum at ∼335 nm for both samples, which probably signifies the presence of an excitonic peak, a representative of 2D layered materials.^17^ The absorption maximum appeared due to the electronically spin-forbidden ^1^S_0_ → ^3^P_1_ transition of Bi^3+^ ion.^9*a*,*b*^ While previous reports showed no noticeable PL emission at room temperature for Cs_4_CdBi_2_Cl_12_ NCs,^9*a*,*b*^ we have obtained room temperature weak PL emission for the pristine NS sample, which is centred at 605 nm irrespective of the excitation wavelength (Fig. 4a). The peak possibly appears from the ^3^E_g_ → ^1^A_1g_ electronic transition of the Cd^2+^ ion in the [CdCl_6_]^4−^ octahedral unit.^9*b*^ Conversely, an intense PL emission centred at 601 nm was observed for the Mn-substituted NS sample, which also remains unaffected by the different excitation wavelengths (Fig. 4a). This intense orange emission can be ascribed to the ^4^T_1g_ (G) → ^6^A_1g_ (S) electronic transition of Mn^2+^ ions.^9*b*^ The PL emission of Mn-substituted NSs revealed a broad and large Stokes shifted emission without any overlap between its excitation and emission spectra. The large Stokes shifted broad PL emission of the pristine sample indicates that the emission originates from a characteristic STE state, whereas the broadband and Stokes-shifted PL emission of Mn-substituted NSs appears because of the proficient energy transfer from the STE state to the Mn^2+^ centre.^15^ Fig. S11b and c (ESI†) present the PLE spectra (monitored near PL peak emission) of both samples, which displayed nearly overlapped profiles with the corresponding absorption spectra. The quantum yield (QY) of both NS samples (dispersed in toluene) was measured at room temperature. The QY of pristine NSs was found to be 0.27%, whereas a relatively high quantum yield of 20.56% was recorded for the Mn-substituted NS sample. To the best of our knowledge, this is the highest PLQY of any of the 2D NS samples of Pb-free LDPs. Previously, Yang *et al.*^9*b*^ reported a QY of 4.6% for Cs_4_(Cd_1−*x*_Mn_*x*_)Bi_2_Cl_12_ NCs. The PLQY values of other earlier reported LDPs, such as Cs_4_Mn_0.12_Cd_0.88_Sb_2_Cl_12_ NCs, Cs_4_CuIn_2_Cl_12_ NCs, and Cs_4_MnBi_2_Cl_12_ NCs are found to be 3.90%, 1.70%, and <1%, respectively.^18^ Additionally, the PLQY values of earlier reported perovskite halide NS [or nanoplate (NPL)] samples were compared with the PLQY values of our NS sample along with their thickness values (Table S4, ESI†).

To examine the temperature stability of the NS sample, we performed temperature-dependent PL measurement of Mn-substituted NSs dispersed in toluene in the 25–85 °C temperature range. The PL spectra at 85 °C revealed a relative loss of only 26% of its initial intensity (Fig. S12a, ESI†), which can also be witnessed from the appearance of the sample under UV illumination (Fig. S12a,† inset). Furthermore, we added up to 120 μl of water into 3 ml of the NS sample in toluene and monitored the PL with gradual addition of water (Fig. S12b, ESI†). The PL spectra and visual appearance shown in Fig. S12b (ESI†) indicate a 57% relative loss of its initial intensity and a weak orange emission, respectively. These observations again lead to the relatively high temperature- and reasonable water stabilities of the as-synthesized Mn-substituted NS sample and show that this can be a promising candidate for optoelectronic application, comparable to the conventional Pb-based 3D perovskite NCs.

For better understanding of the charge carrier dynamics in these NS samples, PL measurement was performed at cryogenic temperature (77 K). Fig. 4b displays the low-temperature PL spectra for both the pristine and Mn-substituted NSs, respectively. An enhanced PL intensity and red-shifted emission towards the near-infrared (NIR) region were observed for both the samples at 77 K in comparison to room temperature PL. Similar trends in PL with temperature were reported earlier for Cs_3_BiBr_6_ NCs.^19^ The enhanced PL intensity at 77 K for both samples is attributed to the suppression of the thermally activated non-radiative recombination pathways.^20^ The red-shifted PL events for Mn-substituted NSs were originated from the enhanced crystal field strength owing to the lattice contraction at low temperature, which resulted in reduced energy of the ^4^T_1g_ → ^6^A_1g_ transition and a subsequent, red-shifted energy peak.^10^ The suppression of electron–phonon coupling at low temperature might be responsible for the emission line-width narrowing in the Mn-substituted sample.^9*b*^ In contrast, a broader PL signature was witnessed for pristine NSs at 77 K, which have been fitted with a Gaussian line-shape function into two contributions (Fig. S13, ESI†). The low-energy PL contribution is ascribed to the additional recombination from intragap localized states (*i.e.*, shallow-defect states) that cause the unusual spectral broadening.^21^ Such observations were previously reported for Pb-based perovskites.^22^

To obtain insights into the excitonic recombination dynamics, time-resolved PL studies were carried out for both NS samples at 298 K and 77 K, as depicted in Fig. 4c and d. The PL decays of pristine and Mn-substituted NS samples are fitted with the biexponential function. The decay curve can be represented by using the following equation:
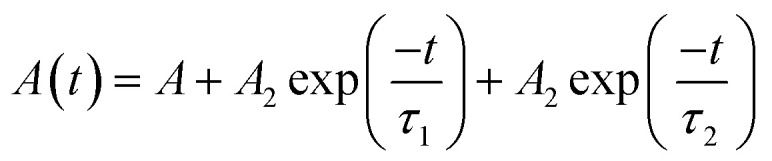
where *A* denotes the emission intensity, and *A*_1_ and *A*_2_ are the fitting parameters describing the relative amplitude for varied lifetime components, while *τ*_1_ and *τ*_2_ are the respective lifetime components. The details of the fitted lifetime parameters with their relative proportions are summarized in Table S5 (ESI†), in which the lifetimes fall in the order of the microsecond time scale due to the forbidden nature of the involved transitions. Notably, the PL decay plots for both NS samples in Fig. 4c and d suggested slower decay dynamics and a prolonged average lifetime (*τ*_avg_) at 77 K than that at 298 K (Table S5, ESI†), which might be due to the higher probability of radiative recombination, and subsequent elimination of non-radiative recombination channels.^9*a*^ Furthermore, to verify the presence of the trap-state contribution at 77 K for pristine NSs, we have measured the lifetime by monitoring at two deconvoluted PL contributions, and a much shorter *τ*_avg_ was achieved when monitored at low energy PL contribution.^9*a*,22*c*^ Previously reported Cs_4_Mn_*x*_Cd_1−*x*_Bi_2_Cl_12_ NC samples also exhibited similar enhancement in the average lifetime at cryogenic temperature compared to that at 300 K.^9*a*,*b*^

**Fig. 5:**
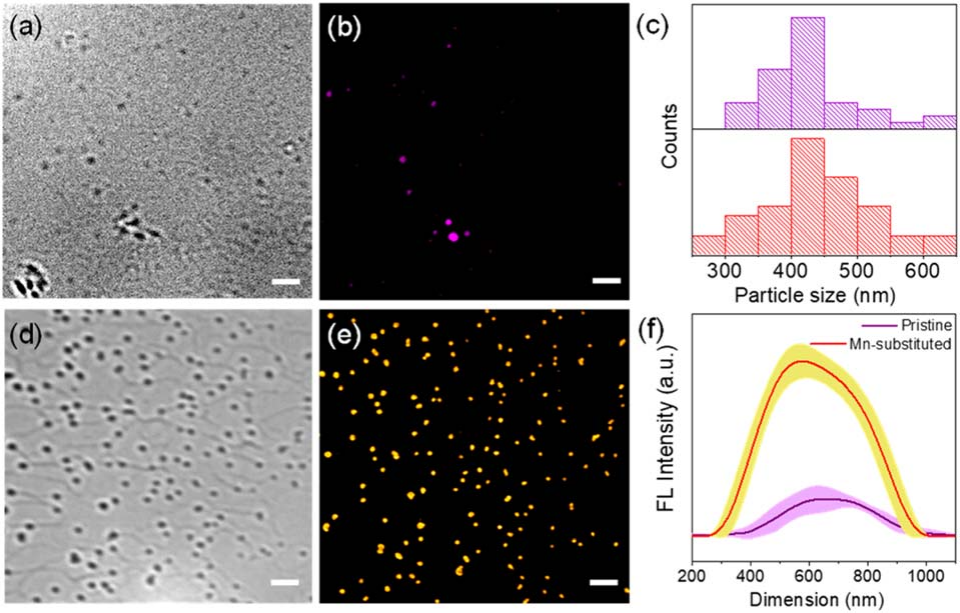
Optical microscopy images for Cs_4_CdBi_2_Cl_12_ (pristine) and Cs_4_Cd_0.6_Mn_0.4_Bi_2_Cl_12_ (Mn-substituted) nanosheet (NS) samples where (a) and (d) are the bright field images, whereas (b) and (e) are the fluorescence microscopy images using the SIM method. Scale bar is 2 μm for all the images. (c) Size distribution histogram for pristine and Mn-substituted NS samples. (f) The average fluorescence intensity plot of pristine and Mn-substituted NS samples from the SIM images captured with the same microscopy parameters. We picked 5 representative NSs from each sample and calculated intensity profiles for each of these NSs. The error bar data show the variations of intensity of these selected NSs.

In order to investigate the PL properties of individual single crystalline NSs, we acquired fluorescence images *via* super-resolved structured illumination microscopy (SIM) after depositing a diluted solution of NSs onto a glass coverslip. Fig. 5a and d show the bright-field optical images of the NSs, where spatially separated NSs can be clearly observed on the glass coverslip. In order to acquire fluorescence images from these NSs, a 405 nm laser was employed as the excitation source for the super-resolution SIM imaging study at the single-particle level. A band-pass filter collected the emission in an optical window of 575–650 nm. The fluorescence images from the NS samples, obtained by structured illumination microscopy (SIM), are shown in Fig. 5b and e. The SIM image typically looked crisper with improved resolution (see Fig. S14 and S15, ESI†) as compared to a standard wide-field image. It is evident from the SIM images that because of the high PLQY of the Mn-substituted NS, the spatially separated particles of the Mn-substituted analogue could be readily visualized with high contrast. In comparison, the pristine NS sample, although visible, appeared much dimmer compared to the Mn-substituted NS sample. The particle size histogram from SIM images (Fig. 5c) revealed a distribution of NSs with sizes ranging from 300–600 nm. The size distributions of the perovskite nanosheets (NSs), presented in Fig. 5c, have been determined by using the fluorescence intensity profiles of the NSs. Rapid scanning and large area sampling capability of fluorescence microscopy helped in quick determination of the size distribution profile of the NSs. From the SIM images (Fig. S15, ESI†), we first plotted the cross-sectional intensity profile of the individual NS. The full width at half maximum (FWHM) from the Gaussian fit of this cross-sectional profile was reported as the size of the NSs. In an unbiased fashion, we have picked ∼40 particles from the imaging window and measured the FWHM for determining the size distribution of the NSs. In this respect it should be noted that the typical achievable lateral (*xy*) resolution of SIM is around 100–120 nm. On the other hand, from our image analysis we found that even the smaller size NSs were >200 nm (Fig. 5c). This indicates that the size of NSs was well above the resolution limit of the SIM methods of imaging. Therefore, we believe that in this case, SIM images are capable of providing near accurate size estimation and it is comparable to the data obtained from the TEM analysis (Fig. 3b and c). Importantly, given that the average size from fluorescence images (∼400 nm) matches with that from other techniques (*e.g.*, TEM), it ruled out the possibility of analysis being performed on aggregated NSs. In addition, a quantitative estimation from the fluorescence intensity plot in Fig. 5f clearly reflects a considerably higher average intensity from the Mn-substituted NSs compared to the pristine NSs. Moreover, we have also calculated the PL intensity *vs.* particle size data for a large number of NSs of the pristine and Mn-substituted samples. These data have been represented in a scatter plot in Fig S16,† which also indicated a considerably large fluorescence intensity in the case of Mn-substituted NSs with similar size particles.

**Fig. 6:**
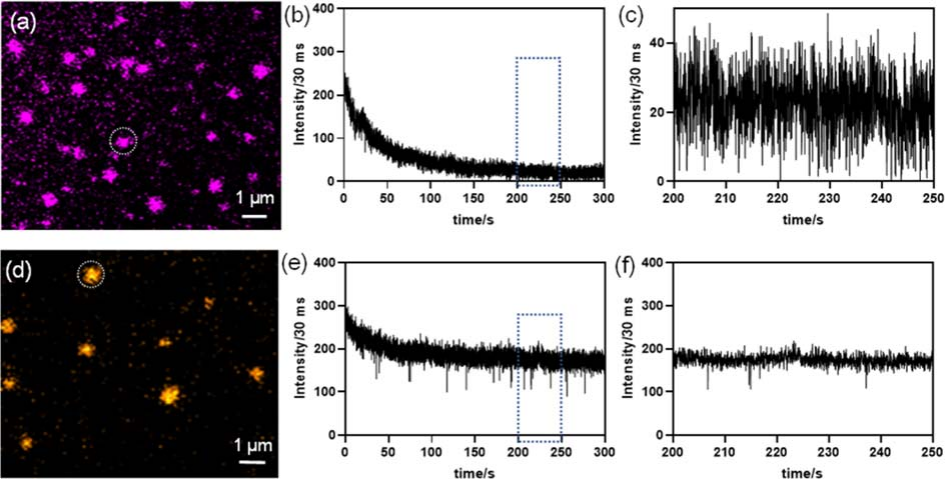
Time dependent fluorescence time traces (b) and (e) for nanosheets (NSs) of Cs_4_CdBi_2_Cl_12_ (pristine) and Cs_4_Cd_0.6_Mn_0.4_Bi_2_Cl_12_ (Mn-substituted) were generated from the NS circled in the diffraction limited images (a) and (d), respectively. (c) and (f) are zoomed-in time trace plots for 50s [from the region shown by the blue rectangle in (b) and (e)] depicting the different PL fluctuation behaviours of pristine and Mn-substituted NSs.

Finally, to acquire deeper insights into the PL characteristics of the pristine and Mn-substituted NSs, we investigated temporal instability of PL from individual single crystalline NSs under continuous excitation. In general, the nature of time-dependent PL intermittency (or “blinking”) is dependent on the metastable non-radiative recombination sites.^23^ This further results in a low-emissive dark (OFF) state or quenched PL state (active), whereas a smaller number of such traps provides an enhanced photo-stability in the intense high emissive (ON) state or bright PL state (in-active).^23,24^ These low-emissive states in blinking events are a major setback in wide-ranging applications, such as in LEDs and biological settings for tracking single biomolecules.^24*a*^ Therefore, it is important to characterize the temporal instability of PL behaviour of the as-synthesized NSs under constant illumination. For the fluorescence time-trace analysis, we continuously collected emission (video) over 100 s for pristine and Mn-substituted NS samples individually under the total internal reflection fluorescence (TIRF) setting (videos S1 and S2, respectively, ESI†). Fig. 6a and d depict the representative NSs that were considered for the temporal PL instability study in the respective samples. The video snapshots from the respective samples under TIRF at an interval of 15 s are shown in Figs. S17a and b (ESI†). The pristine NS exhibited a rapid reduction in luminescence intensity in the PL *versus* time-trace plot; however, such behaviour was not observed in the Mn^2+^-substituted NS sample (Fig. 6b and e, respectively). After an initial decrease in intensity due to the illumination induced photo-bleaching, the zoomed-in time trace plot in Fig. 6c reveals that the pristine NS tends to display a PL blinking-like behaviour with a number of OFF states after 200 s exposure to continuous laser radiation. This blinking-like nature can be presumably ascribed to the presence of trap states that results in the OFF state.^25^ This is further supported by the time-resolved PL decay at room temperature, where nearly 50% contribution in the life-time originates from the faster component (*τ*_1_), which resulted from the trap state contribution (Table S3, ESI†).

Interestingly, from the time-dependent PL traces up to 120 s in Fig. 6e and the recorded video (video S2, ESI†) for Mn-substituted NSs, no significant decrease in luminescence intensity from PL-*versus*-time traces was observed from a single particle. The PL intensity is found to slightly fluctuate between the high intensity ON states, which is well-above the background intensity level. A zoomed in plot within 50 s regimes (after the initial 200 s exposure) further confirms the absence of the non-zero OFF state and a distinctly suppressed PL fluctuation nature (Fig. 6f). The PL intensity traces found at the single NS level established that the inclusion of Mn^2+^ in pristine NSs (*i.e.*, Mn-substituted analogue) can effectively arrest the dark OFF-state. Such a rare behaviour with suppressed PL fluctuations without any photo-bleaching event was observed earlier for halide perovskite-semiconductor core/shell quantum dots,^24*a*^ CsPbI_3_ NCs when NH_4_I was used as the precursor,^26^ MAPbX_3_ QDs (X = Br, I) using a bromide or iodide donor (MABr and MAI) as a filler,^27^ and Yb^3+^- and Er^3+^-doped up-converting NaYF_4_ NCs.^28^

The weak PL emission in pristine NSs is induced by the dynamic equilibrium between active and in-active states of the metastable non-radiative recombination channels, causing PL fluctuations or a blinking-like nature. However, in partially Mn-substituted NSs, the PLQY is much higher, which means the number of non-radiative centers is much smaller, but we still see some PL fluctuation like events. Similar defects are probably still present in Mn-substituted NSs; however, their number is much less, and these non-radiative centers are mostly in the passive state. Thereby, partial Mn^2+^ substitution seems to stabilize the in-active state of the non-radiative centers, which leads to an intense PL emission, and seldom and short-lived off states.

## Conclusions

Facile solvent-free mechanochemical synthesis and optical property investigations have been demonstrated for the bulk powder of pristine and its partially Mn-substituted analogue. Importantly, a modified hot-injection route was implemented to synthesize the 2D NSs of pristine and Mn-substituted analogue. A weak PL emission is perceived for pristine NSs at room temperature with a QY of <1%. Conversely, the Mn-substituted NSs showed an intense orange emission at room temperature together with a QY of ∼21%. The steady-state and time-resolved PL measurements at cryogenic temperature (77 K) revealed the involvement of trap states in weak PL emission of the pristine sample, which is substantially reduced by the addition of Mn^2+^ in partially Mn-substituted NSs, resulting in an intense PL. Finally, super resolution fluorescence microscopy was implemented to understand the single nanosheet's PL properties in the pristine NSs and the Mn-substituted analogue. The pristine NS exhibited illumination induced photo-bleaching and PL blinking-like nature, whereas the Mn-substituted NS analogue demonstrated suppression of PL fluctuations with negligible photo-bleaching behaviour. The PL intensity traces with time at the single NS level established that the introduction of Mn^2+^ in pristine NSs can stabilize the in-active state of the metastable non-radiative channels. Characterized by an intense orange emission with suppressed PL fluctuations and negligible photo-bleaching, the Mn-substituted NSs demonstrate their unique optical properties at the single NS level, which might be utilized for further optoelectronic applications in the future.

## Data availability

All data are available in the manuscript and in the ESI.†

## Author contributions

K. B. conceived the idea and designed the study. A. B., K. K., P. A., J. P. and K. B. carried out the synthesis, structural and optical measurements, and other analyses. R. S., S. K. and S. S. A. studied the photoluminescence blinking properties. K. K. wrote the first draft and everyone contributed to editing the manuscript.

## Conflicts of interest

The authors declare no conflict of interest.

## Supplementary Material

SC-014-D3SC02506C-s001

## References

[cit1] Pradhan N. (2021). Acc. Chem. Res..

[cit2] Su P., Liu Y., Zhang J., Chen C., Yang B., Zhang C., Zhao X. (2020). J. Phys. Chem. Lett..

[cit3] Ju M.-G., Dai J., Ma L., Zeng X. C. (2017). J. Am. Chem. Soc..

[cit4] Fan Q., Biesold-McGee G. V., Ma J., Xu Q., Pan S., Peng J., Lin Z. (2020). Angew. Chem., Int. Ed..

[cit5] Tang H., Xu Y., Hu X., Hu Q., Chen T., Jiang W., Wang L., Jiang W. (2021). Adv. Sci..

[cit6] Vargas B., Torres-Cadena R., Reyes-Castillo D. T., Rodríguez-Hernández J., Gembicky M., Menéndez-Proupin E., Solis-Ibarra D. (2020). Chem. Mater..

[cit7] Vargas B., Ramos E., Pérez-Gutiérrez E., Alonso J. C., Solis-Ibarra D. (2017). J. Am. Chem. Soc..

[cit8] Holzapfel N. P., Majher J. D., Strom T. A., Moore C. E., Woodward P. M. (2020). Chem. Mater..

[cit9] Bai T., Yang B., Chen J., Zheng D., Tang Z., Wang X., Zhao Y., Lu R., Han K. (2021). Adv. Mater..

[cit10] Vargas B., Reyes-Castillo D. T., Coutino-Gonzalez E., Sánchez-Aké C., Ramos C., Falcony C., Solis-Ibarra D. (2020). Chem. Mater..

[cit11] Vargas B., Torres-Cadena R., Rodríguez-Hernández J., Gembicky M., Xie H., Jiménez-Mier J., Liu Y.-S., Menéndez-Proupin E., Dunbar K. R., Lopez N., Olalde-Velasco P., Solis-Ibarra D. (2018). Chem. Mater..

[cit12] Manna D., Kangsabanik J., Das T. K., Das D., Alam A., Yella A. (2020). J. Phys. Chem. Lett..

[cit13] Yadav R., Swain D., Kundu P. P., Nair H. S., Narayana C., Elizabeth S. (2015). Phys. Chem. Chem. Phys..

[cit14] Kirillov S. A., Voyiatzis G. A., Musiyenko I. S., Photiadis G. M., Pavlatou E. A. (2001). J. Chem. Phys..

[cit15] Peng C., Wei Q., Chen L., Zeng R., Zhang Q., Hu Q., Zou B. (2021). J. Mater. Chem. C.

[cit16] Murzakhanov F., Mamin G., Voloshin A., Klimashina E., Putlyaev V., Doronin V., Bakhteev S., Yusupov R., Gafurov M., Orlinskii S. (2018). IOP Conf. Ser.: Earth Environ. Sci..

[cit17] Li J., Yu Q., He Y., Stoumpos C. C., Niu G., Trimarchi G. G., Guo H., Dong G., Wang D., Wang L., Kanatzidis M. G. (2018). J. Am. Chem. Soc..

[cit18] Liu M., Matta S. K., Ali-Löytty H., Matuhina A., Grandhi G. K., Lahtonen K., Russo S. P., Vivo P. (2022). Nano Lett..

[cit19] Tang Y., Gomez L., van der Laan M., Timmerman D., Sebastian V., Huang C.-C., Gregorkiewicz T., Schall P. (2021). J. Mater. Chem. C.

[cit20] Sheikh T., Maqbool S., Mandal P., Nag A. (2021). Angew. Chem., Int. Ed..

[cit21] Imran M., Peng L., Pianetti A., Pinchetti V., Ramade J., Zito J., Di Stasio F., Buha J., Toso S., Song J., Infante I., Bals S., Brovelli S., Manna L. (2021). J. Am. Chem. Soc..

[cit22] Delmas W. G., Vickers E. T., DiBenedetto A. C., Lum C., Hernandez I. N., Zhang J. Z., Ghosh S. (2020). J. Phys. Chem. Lett..

[cit23] Merdasa A., Tian Y., Camacho R., Dobrovolsky A., Debroye E., Unger E. L., Hofkens J., Sundström V., Scheblykin I. G. (2017). ACS Nano.

[cit24] Tang X., Yang J., Li S., Liu Z., Hu Z., Hao J., Du J., Leng Y., Qin H., Lin X., Lin Y., Tian Y., Zhou M., Xiong Q. (2019). Adv. Sci..

[cit25] Tian Y., Merdasa A., Peter M., Abdellah M., Zheng K., Ponseca C. S., Pullerits T., Yartsev A., Sundström V., Scheblykin I. G. (2015). Nano Lett..

[cit26] Paul S., Samanta A. (2022). J. Phys. Chem. Lett..

[cit27] Chouhan L., Ito S., Thomas E. M., Takano Y., Ghimire S., Miyasaka H., Biju V. (2021). ACS Nano.

[cit28] Ostrowski A. D., Chan E. M., Gargas D. J., Katz E. M., Han G., Schuck P. J., Milliron D. J., Cohen B. E. (2012). ACS Nano.

