# Supplementary material for: 2D nanosheets of layered double perovskites: synthesis, photostable bright orange emission and photoluminescence blinking
Source: Chem Sci. 2023 Jun 20;14(26):7161–9. doi: 10.1039/d3sc02506c (PMC10321497; doi:10.1039/d3sc02506c)
Supplement: SC-014-D3SC02506C-s001 [file SC-014-D3SC02506C-s001.pdf]

Electronic Supplementary Material (ESI) for Chemical Science.
This journal is © The Royal Society of Chemistry 2023

Electronic Supplementary Information (ESI)

2D Nanosheets of Layered Double Perovskites: Synthesis, Photostable Bright Orange Emission and Photoluminescence Blinking

Aditya Bhardwaj,[a, ‡] Kaushik Kundu,[a, ‡] Ranjan Sasmal,[a] Paribesh Acharyya,[a] Jayita Pradhan,[a] Simanta Kalita,[b] Sarit S. Agasti,[*,a,b] and Kanishka Biswas*[,a]

[a]New Chemistry Unit and School of Advanced Materials Jawaharlal Nehru Centre for Advanced Scientific Research (JNCASR), Jakkur P.O., Bangalore 560064, India.

* E-mail: sagasti@jncasr.ac.in; *E-mail: kanishka@jncasr.ac.in

[b]Chemistry and Physics of materials Unit, JNCASR, Jakkur P.O., Bangalore 560064, India.

[‡] These authors contributed equally to this work

EXPERIMENTAL SECTION

Reagents. Cesium carbonate (Cs_2CO_3, 99.9% Sigma Aldrich), bismuth acetate [$Bi(OAc)_3$, >99.99% Sigma Aldrich], manganese acetate [$Mn(OAc)_2$,> 98% Sigma-Aldrich], cadmium acetate dihydrate [$Cd(OAc)_2 \cdot 2H_2O$, 98% Alfa Aesar], manganese chloride ($MnCl_2$, >99% Sigma Aldrich), cesium chloride (CsCl, >99.99% Sigma Aldrich), bismuth chloride ($BiCl_3$,>99.99% Sigma Aldrich), cadmium chloride ($CdCl_2$, >99.99% Sigma-Aldrich trace metal basis), benzoyl chloride (ACS reagent, 99%, Sigma-Aldrich), 1-octadecene (ODE, technical grade, 90%), oleylamine (OAm, technical grade, 90%), oleic acid (OA, technical grade, 90%), toluene (ACS reagent, ≥99.5%, Sigma-Aldrich), and isopropanol (ACS reagent, ≥99.5%, Sigma-Aldrich) were used as received without further purification.

Synthesis of $Cs_4CdBi_2Cl_{12}$ and $Cs_4Cd_{0.6}Mn_{0.4}Bi_2Cl_{12}$ by solid-state mechanochemistry. Precisely, 135.8 mg (0.8 mmol) of CsCl, 37.0 mg (0.2 mmol) of $CdCl_2$, 127.2 mg (0.4 mmol) $BiCl_3$ were grounded mechanically for 2 hours in a mortar pestle to synthesize $Cs_4CdBi_2Cl_{12}$. Similarly, 138 mg (0.8 mmol) of CsCl, 22.5 mg (0.12 mmol) of $CdCl_2$, 129.2 mg (0.4 mmol) $BiCl_3$, 10.3 mg (0.08 mmol) of $MnCl_2$ for were grounded together for the synthesis of $Cs_4Cd_{0.6}Mn_{0.4}Bi_2Cl_{12}$. The synthesis were carried out at ambient conditions.

Colloidal synthesis of nanosheets (NSs) of $Cs_4CdBi_2Cl_{12}$ and $Cs_4Cd_{0.6}Mn_{0.4}Bi_2Cl_{12}$. In a typical synthesis, 65.16 mg (0.2 mmol) of Cs_2CO_3, 26.65 mg (0.1 mmol) of $Cd(OAc)_2.2H_2O$, and 77.22 mg (0.2 mmol) of $Bi(OAc)_3$ for $Cs_4CdBi_2Cl_{12}$; and 65.16 mg (0.2 mmol) of Cs_2CO_3, 15.99 mg (0.06 mmol) of $Cd(OAc)_2.2H_2O$, 6.92 mg (0.04 mmol) of $Mn(OAc)_2$, and 77.22 mg (0.2 mmol) of $Bi(OAc)_3$ for $Cs_4Cd_{0.6}Mn_{0.4}Bi_2Cl_{12}$ were taken in 10 ml of ODE into a 50 ml tri-necked round bottom flask in Schlenk line. The sealed flask was then allowed to vacuum at 70^0C with continued stirring for 30 mins. Then, 1 ml of OA and 0.5 ml of OAm were injected. Subsequently, the temperature was raised to 120°C and kept for 1 hour in N_2 environment. After that, temperature of the reaction was raised to 135°C and 1 ml of benzoyl chloride was quickly injected into the reaction mixture. The reaction was then immediately quenched in an ice water bath. The precipitates were extracted *via* centrifuging at 8000 rpm for 10 mins after which the solution was washed several times with isopropanol, which was then dispersed in toluene for further studies. The reaction scheme has been shown in Scheme S1.

Powder X-Ray diffraction. Rigaku Smart Lab diffractometer was used for collecting the PXRD patterns of all samples at room temperature using Cu Kα radiation (λ =1.5406 Å) with accelerating voltage of 40 kV (current of 30 mA).

Inductively coupled plasma atomic emission spectroscopy (ICP-AES). The exact composition of the as-synthesized nanomaterials was measured based on ICP-AES data. The ICP-AES measurements were carried out using Perkin-Elmer Optima 7000DV instrument. The ICP-AES measurement was carried out by dissolving the powdered nanomaterials in aqua regia (HNO_3: HCl = 1:3) followed by diluting with millipore water. The Cs standard (1000 mg/L, Sigma-Aldrich), Cd standard (1000 mg/L, Sigma-Aldrich), Mn standard (1000 mg/L, Sigma-Aldrich), and Bi standard (1000 mg/L, Sigma-Aldrich) were utilized to determine the compositions in ICP. For the particular measurement, the error bar lies below 1.5 %.

Transmission electron microscopy (TEM). The TEM images of the synthesized $Cs_4CdBi_2Cl_{12}$ and $Cs_4Cd_{0.6}Mn_{0.4}Bi_2Cl_{12}$ nanostructures were acquired using a JEOL (JEM3010) TEM instrument (200 kV accelerating voltage) fitted with a Gatan CCD camera. Subsequently, the obtained images were analysed using the ImageJ software.

Thermogravimetric analysis (TGA). TGA was carried out using 2 STAR TGA instrument. Samples were heated at a rate of 5 °C min^{-1} in the temperature range of 100-800 °C and in the N_2 atmosphere (40 mL min^{-1}).

Raman Spectroscopy. The Raman spectra were collected from Jobin-Yvon Horiba LabRAM HR evolution Raman spectrometer in backscattering geometry with 1800 gr/mm grating and Peltier cooled CCD detector using the excitation source of 514.5 nm (green laser) and 10s of acquisition time.

Field emission scanning electron microscopy (FESEM). A ZEISS Gemini SEM – Field emission scanning electron microscope was implemented to extract the FESEM images. Elemental mapping was carried out during FESEM imaging.

Electronic spectroscopy. The UV-Vis absorption spectra were measured using a PerkinElmer, Lambda-900 UV/vis/near-IR spectrometer. The diffuse reflectance measurement was performed in 250-800 nm range in the reflectance mode for obtaining optical bandgap.

The PL emission spectra of all samples in solid state as well as in solution phase (dispersed in toluene) and the excitation spectra were measured by PerkinElmer LS 55 luminescence spectrometer at different temperatures (25-85^{0}C).

Low-temperature PL measurements. Edinburgh FLS1000 spectrofluorometer was implemented for low-temperature PL measurements at 77 K using a vacuum liquid-nitrogen cryostat.

Lifetime measurements. The time-resolved PL decay plots were collected by using Edinburgh FLS1000 spectrofluorometer coupled with pulsed xenon microsecond flash-lamp at 298 K and 77 K.

X-ray photoelectron spectroscopy. XPS measurement was performed using Kratos Axis Ultra DLP instrument that houses the Al K alpha (1486.6eV) radiation with spot size of 300 µm × 700 µm. All XPS signals were calibrated by setting the Carbon 1s signal to 284.8 eV.

Atomic force microscopy (AFM). AFM measurement was carried out using a Bruker Innova Microscope in tapping mode containing antimony doped Silicon tip and data was analysed with NanoScope software.

Fourier transform infrared spectroscopy (FTIR). FTIR spectra was recorded in the range of 400-4000 cm^{-1} with the utilization of Bruker Optics Alpha-P FTIR spectrophotometer equipped with an attenuated total reflectance (ATR) module.

Electron paramagnetic resonance (EPR). EPR spectra were taken for solid sample in JES-X320 instrument (using X-band microwave ES-11030MWU JEOL Resonance).

Super-resolved fluorescence imaging and time-resolved single particle tracking. The toluene-dispersed samples were drop casted on coverslip at certain concentrations and were left to dry in atmospheric conditions. ZEISS ELYRA PS1 system was used for fluorescence microscopy of nanosheet samples. It makes use of Plan-Apochromat 100x/1.46 Oil DIC M27 objective. The bright field and fluorescence microscopy were taken for the visualization of both the samples (i.e., $Cs_4CdBi_2Cl_{12}$ and $Cs_4Cd_{0.6}Mn_{0.4}Bi_2Cl_{12}$). Structured illumination microscopy (SIM) method with excitation using 405 nm laser, appropriate emission filter (BP 575–650 + LP 755) and CMOS camera were used for fluorescence microscopy. The entire experiment was performed with a laser power density of 13.7 W.cm^{-2}. Second, the blinking studies of individual nanosheet sample were carried out using total

internal reflection (TIRF) mode. The time-dependent PL emission for NSs was recorded for 10000 frames with integration time of 30 ms. Laser power density was kept 37.8 $W.cm^{-2}$ throughout the blinking study. Electron multiplication (EM) gain was kept at 50 in the experiment. The time trace of PL emission and photon counts were evaluated by using ZEN 2.0 software. ImageJ software was used for image processing and other calculations on the nanosheet's dimensions. The data in TIRF mode is acquired under very high-power laser illumination. As a quantitative comparison, the excitation power was 13.7 $W.cm^{-2}$ for the SIM imaging, whereas 37.8 $W.cm^{-2}$ was used for acquiring image. Due to the use of high-power laser illumination the background increased. It is believed that the increase in background fluorescence contributed to the appearance of very small size spots in the imaging field of view. Therefore, we concentrated our investigation on much brighter and large size spots that are observed during SIM imaging.

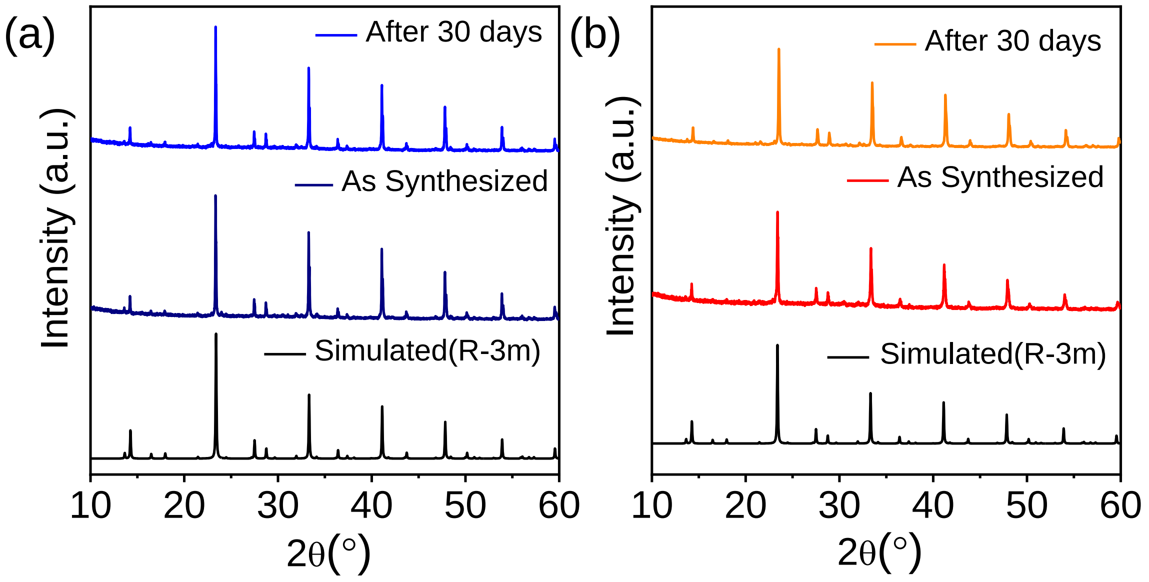

Fig. S1. PXRD pattern of mechanochemically synthesized **(a)** $Cs_4CdBi_2Cl_{12}$, and **(b)** $Cs_4Cd_{0.6}Mn_{0.4}Bi_2Cl_{12}$ bulk powders after 30 days exposure to ambient conditions.

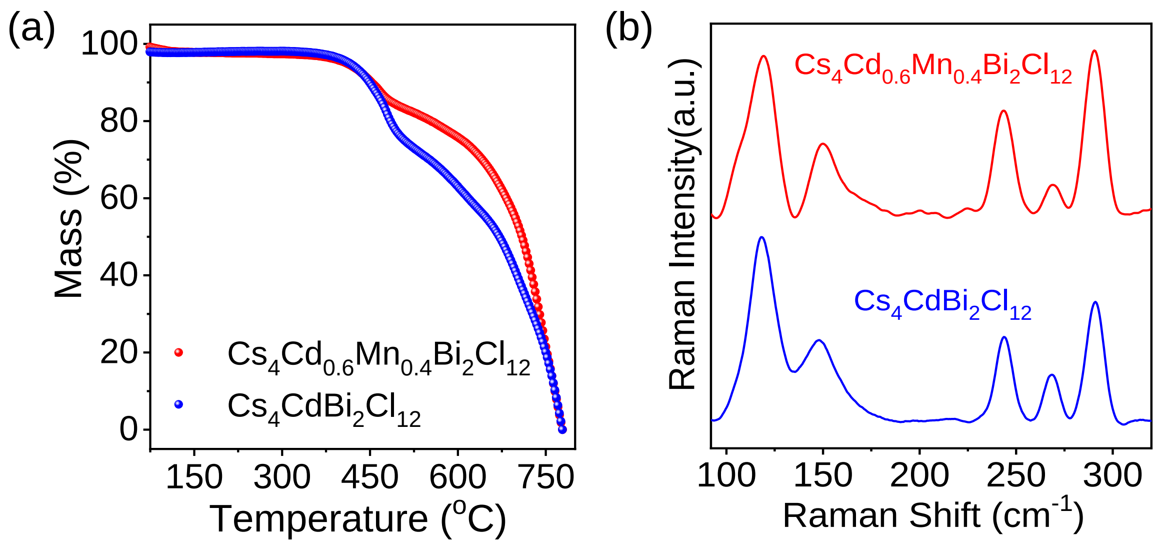

Fig. S2. (a) Thermogravimetric analysis (TGA), and **(b)** Raman spectra of $Cs_4CdBi_2Cl_{12}$ and $Cs_4Cd_{0.6}Mn_{0.4}Bi_2Cl_{12}$ powder samples.

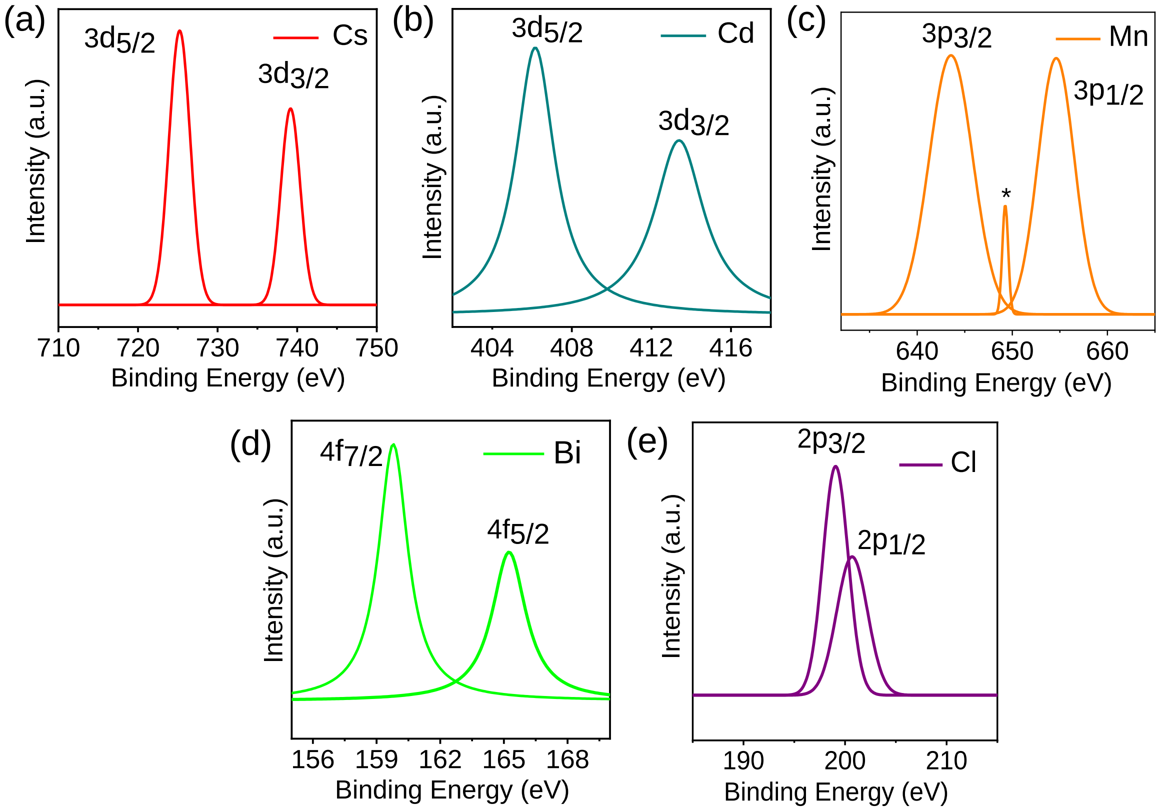

Fig. S3. XPS spectra of $Cs_4Cd_{0.6}Mn_{0.4}Bi_2Cl_{12}$ bulk powders for **(a)** Cs 3d, **(b)** Cd 3d, **(c)** Mn 2p, **(d)** Bi 4f, and **(e)** Cl 2p. The asterisk in (c) denotes the satellite peak in case of Mn^{2+}.

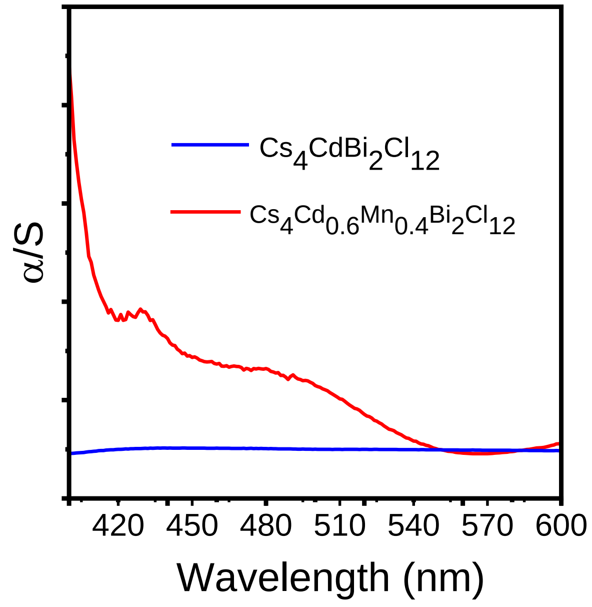

Fig. S4. Zoomed version of solid-state UV/Vis absorption spectra of $Cs_4CdBi_2Cl_{12}$ and $Cs_4Cd_{0.6}Mn_{0.4}Bi_2Cl_{12}$ bulk powders.

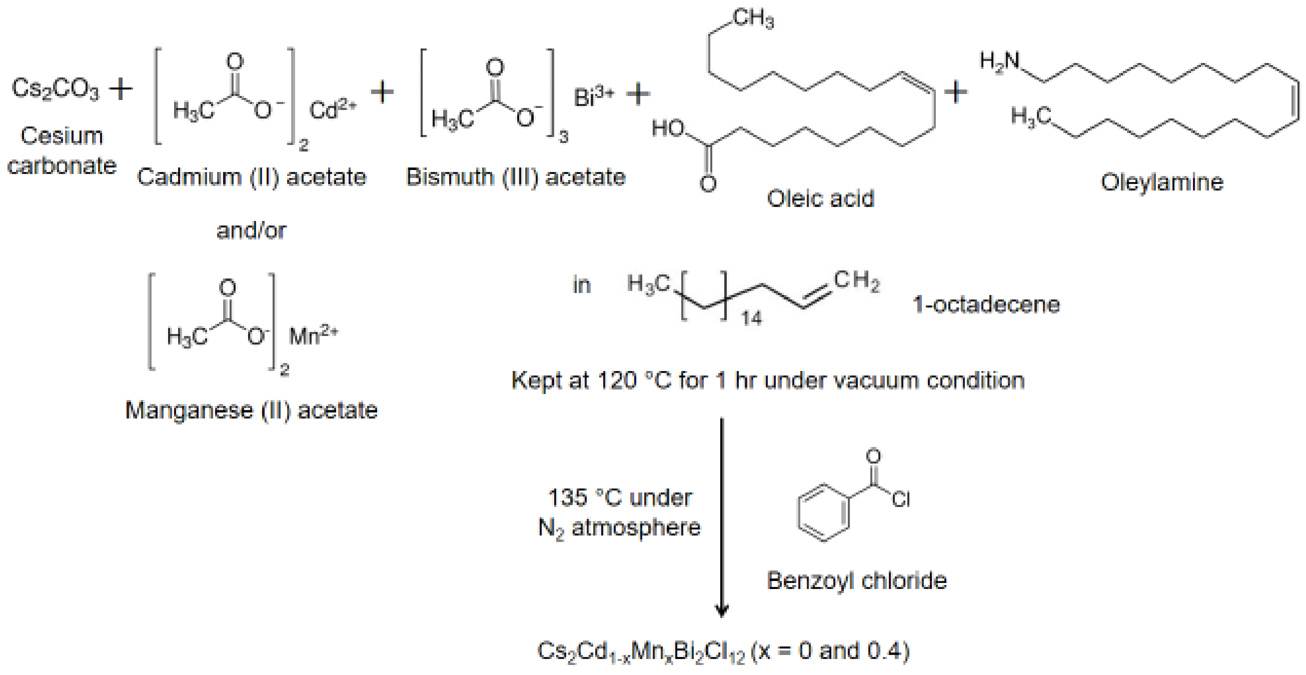

Scheme S1. Detailed colloidal synthesis scheme for nanostructured $Cs_4Cd_{1-x}Mn_xBi_2Cl_{12}$ (x = 0 and 0.4) samples through solution phase hot-injection method.

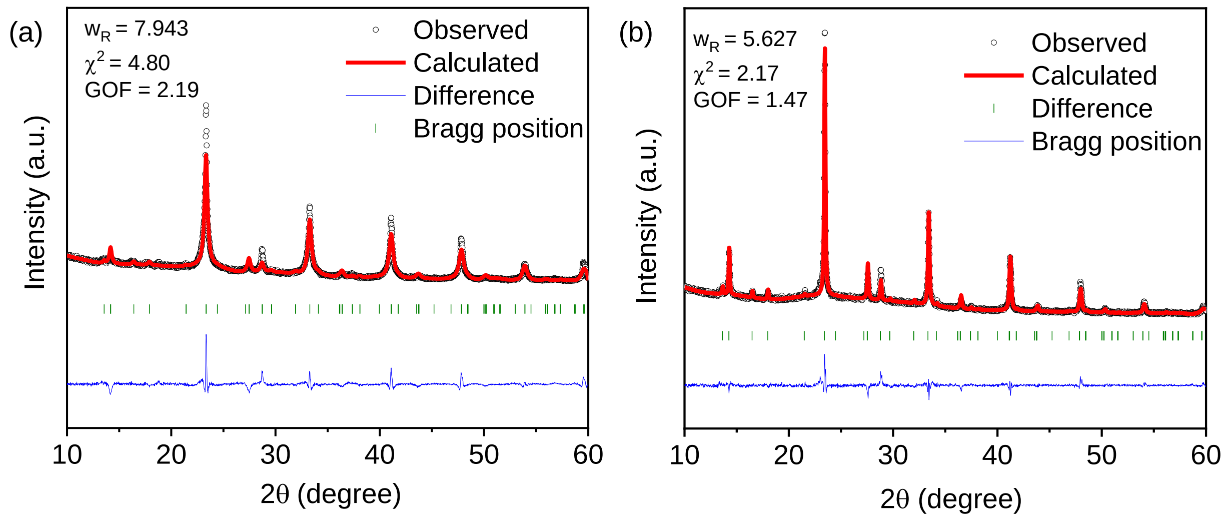

Fig. S5. Rietveld refinement of **(a)** $Cs_4CdBi_2Cl_{12}$, and **(b)** $Cs_4Cd_{0.67}Mn_{0.33}Bi_2Cl_{12}$ nanosheet (NS) samples.

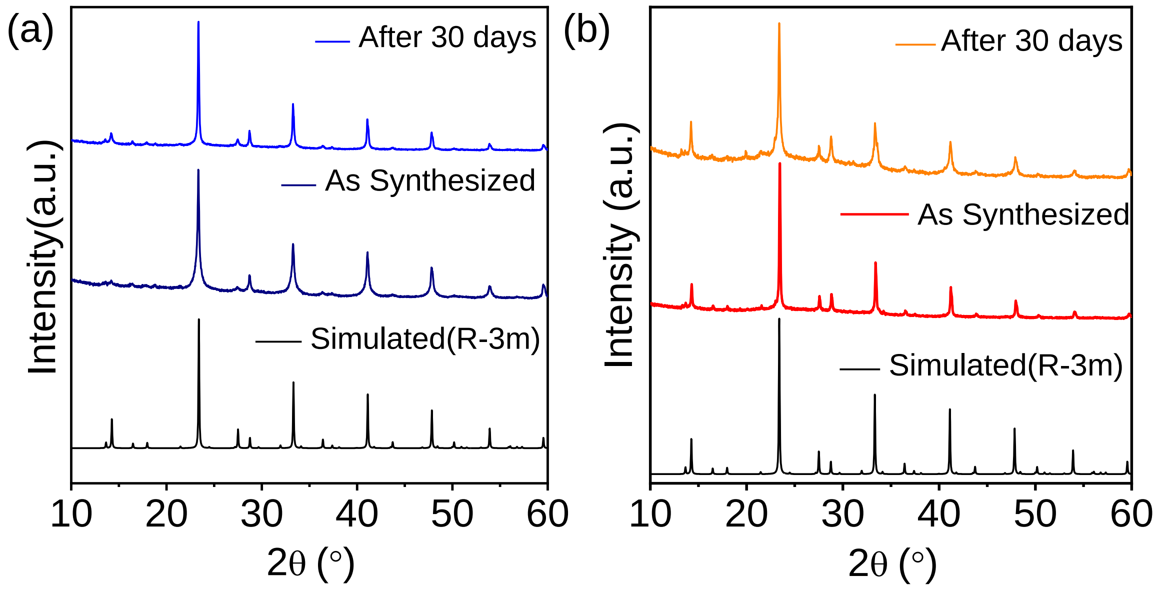

Fig. S6. PXRD pattern of hot-injection synthesized **(a)** $Cs_4CdBi_2Cl_{12}$, and **(b)** $Cs_4Cd_{0.6}Mn_{0.4}Bi_2Cl_{12}$ nanosheet samples after 30 days exposure to ambient conditions.

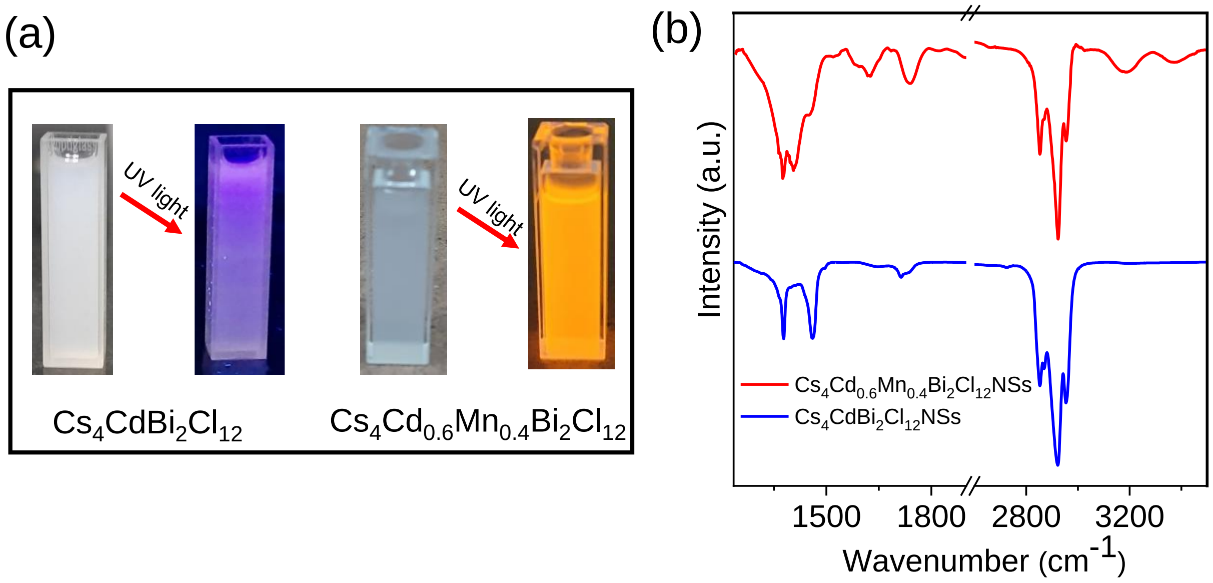

Fig. S7. (a) Physical appearances of nanosheet (NS) samples dispersed in toluene under ambient conditions and inside the UV chamber. **(b)** FTIR spectra of $Cs_4CdBi_2Cl_{12}$ and $Cs_4Cd_{0.6}Mn_{0.4}Bi_2Cl_{12}$ samples synthesized by hot-injection method.

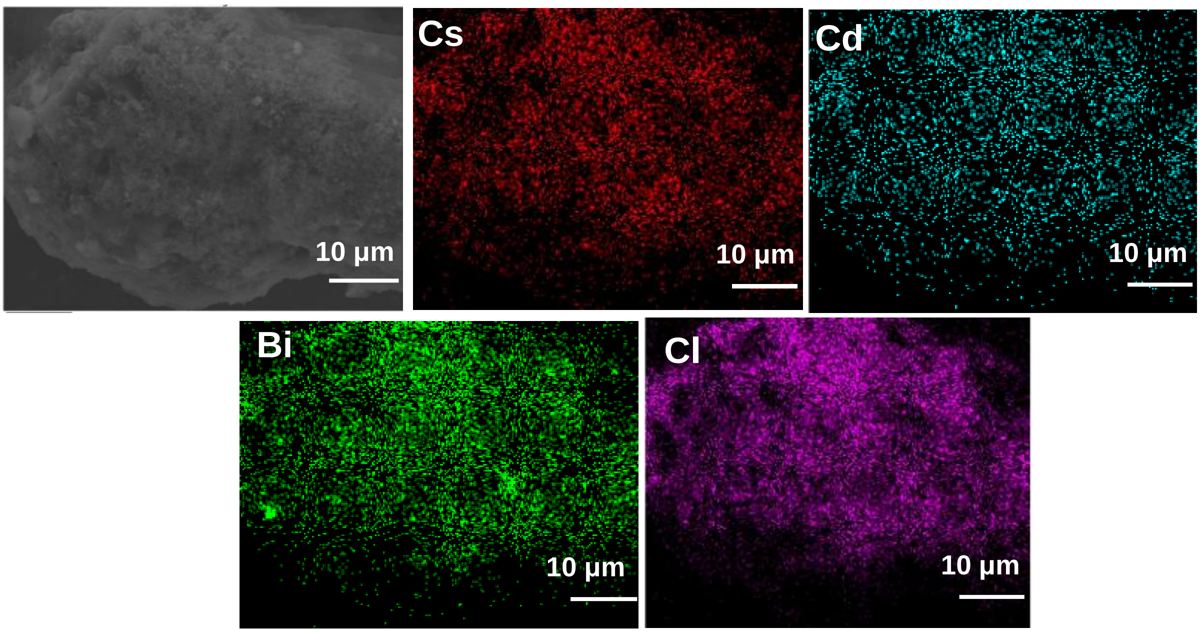

Fig. S8. FESEM image of $Cs_4CdBi_2Cl_{12}$ sample and corresponding elemental colour mapping through EDAX.

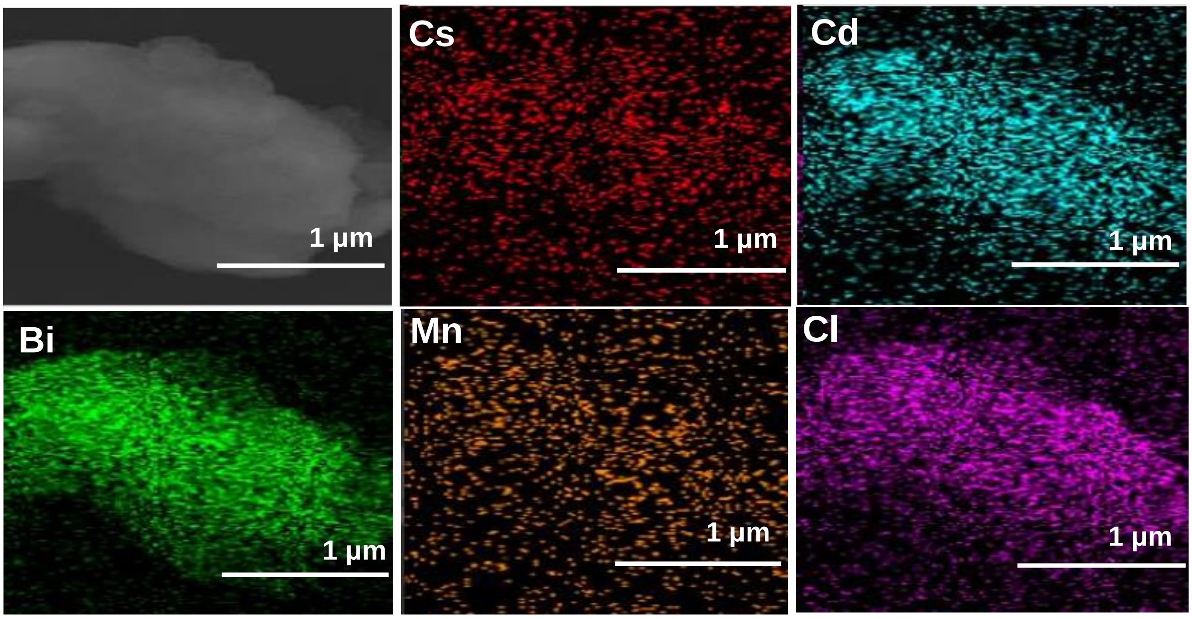

Fig. S9. FESEM image of $Cs_4Cd_{0.6}Mn_{0.4}Bi_2Cl_{12}$ sample and corresponding elemental colour mapping through EDAX.

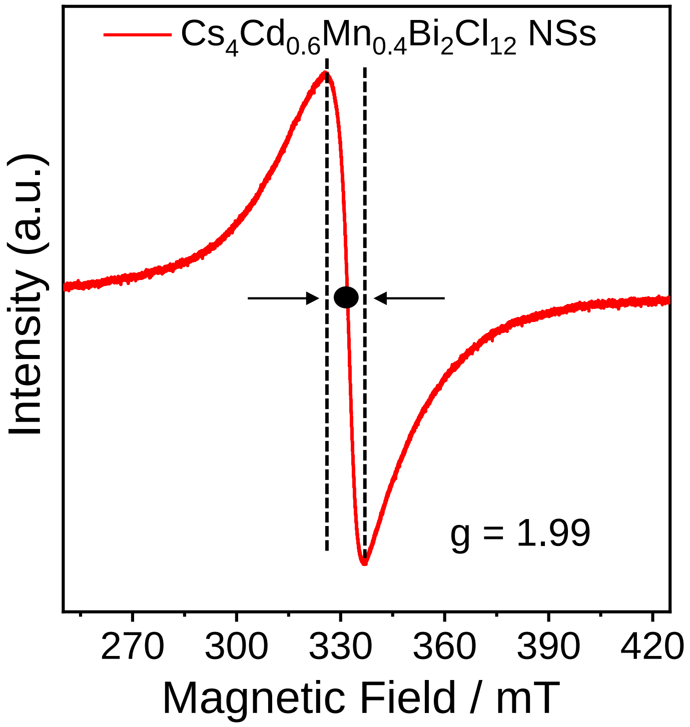

Fig. S10. EPR spectra of hot-injection synthesized $Cs_4Cd_{0.6}Mn_{0.4}Bi_2Cl_{12}$ NS sample.

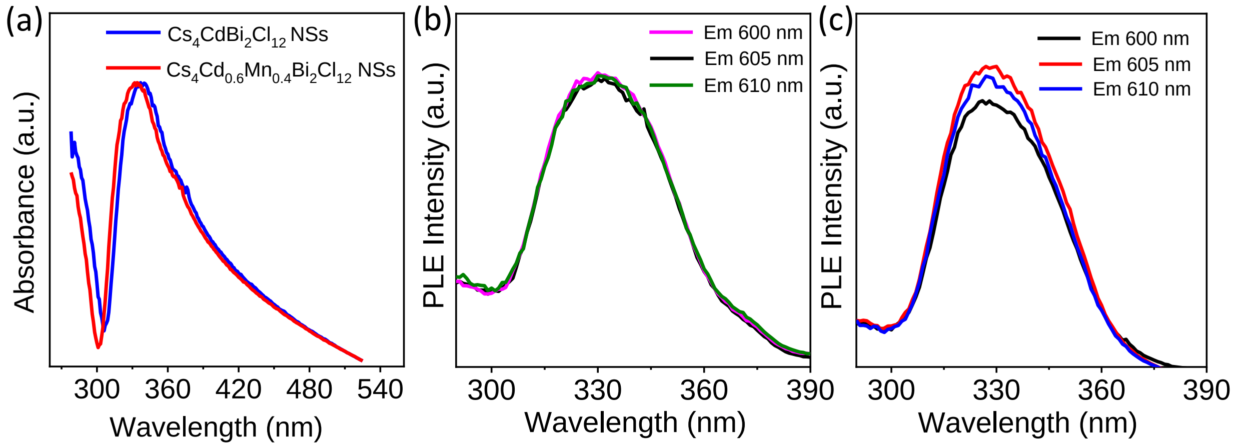

Fig. S11. (a) Electronic absorption spectra of $Cs_4CdBi_2Cl_{12}$ and $Cs_4Cd_{0.6}Mn_{0.4}Bi_2Cl_{12}$ nanosheets (NSs). The emission wavelength dependent PL excitation (PLE) spectra of **(b)** $Cs_4CdBi_2Cl_{12}$, and **(c)** $Cs_4Cd_{0.6}Mn_{0.4}Bi_2Cl_{12}$ nanosheets (NSs).

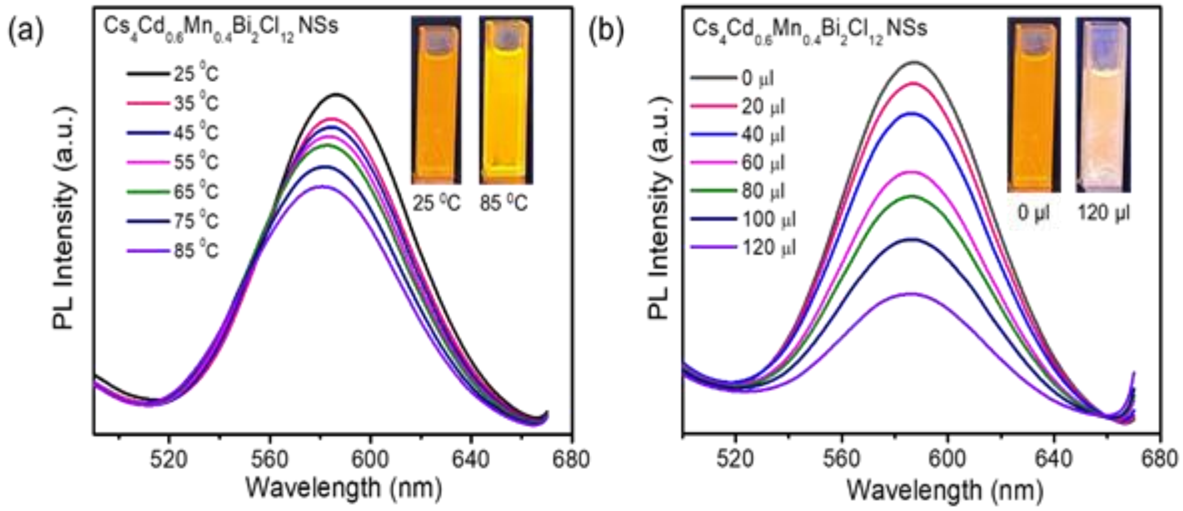

Fig. S12. (a) Temperature dependent PL spectra of $Cs_4Cd_{0.6}Mn_{0.4}Bi_2Cl_{12}$ nanosheets (NSs). **(b)** The change in PL intensity of $Cs_4Cd_{0.6}Mn_{0.4}Bi_2Cl_{12}$ NSs with addition of water. The inset in the respective plots shows the physical appearance of NS solution before and after the study.

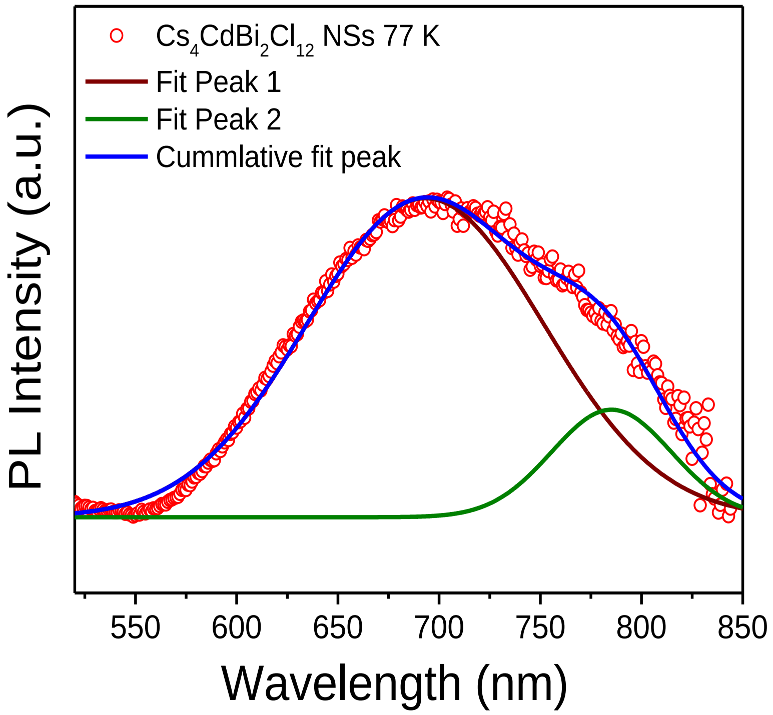

Fig. S13. Deconvoluted PL spectra of $Cs_4CdBi_2Cl_{12}$ nanosheets (NSs) at 77 K.

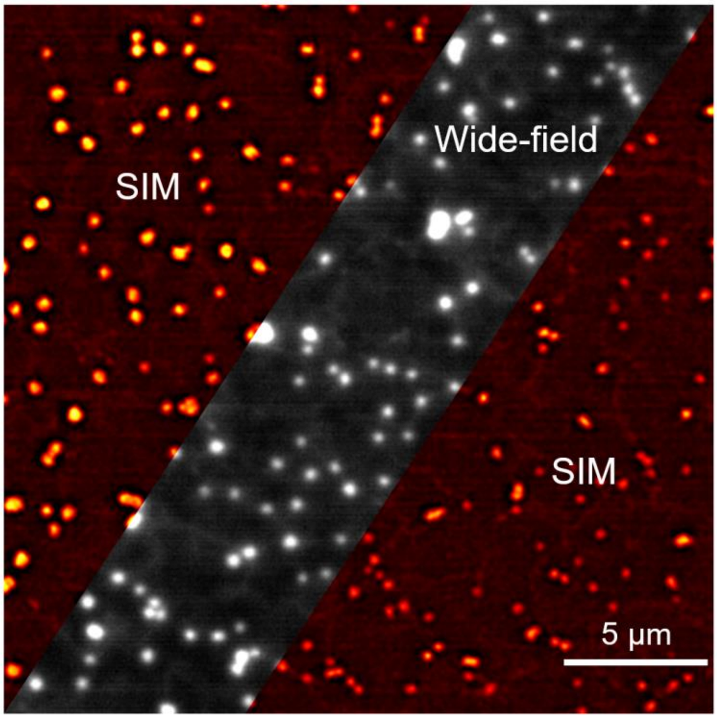

Fig. S14. Comparison between SIM and corresponding wide-field fluorescence image of the Mn-substituted nanosheets.

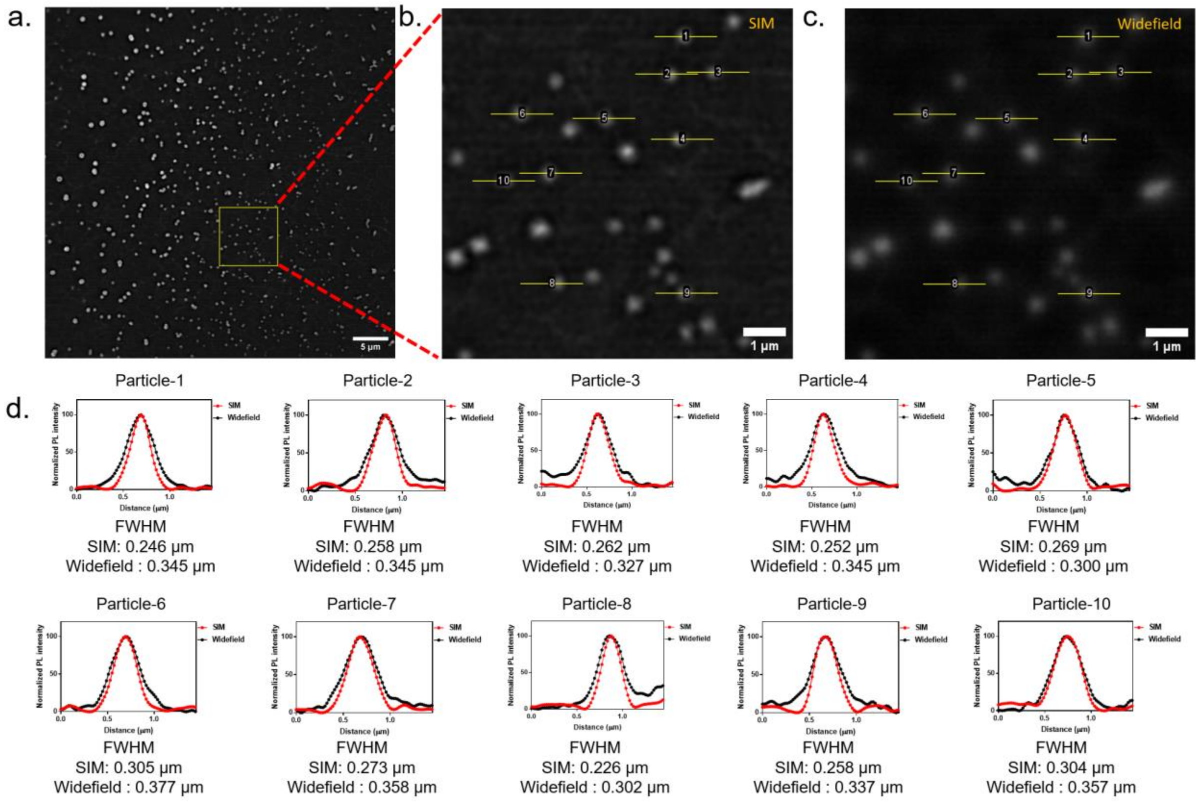

Fig. S15. a) Image of the Mn-substituted nanosheets. b) Zoomed in SIM image of the highlighted yellow square region from image a. c) Corresponding widefield image of the highlighted yellow square region from image a. d) Comparison of sizes of the particles in the SIM and widefield image. The plots are generated by plotting the intensity values from the representative 10 highlighted particles (yellow lines in image b and c). The full width at half maxima (FWHM) from the Gaussian fit of this cross-sectional profile was reported as the size of the NSs.

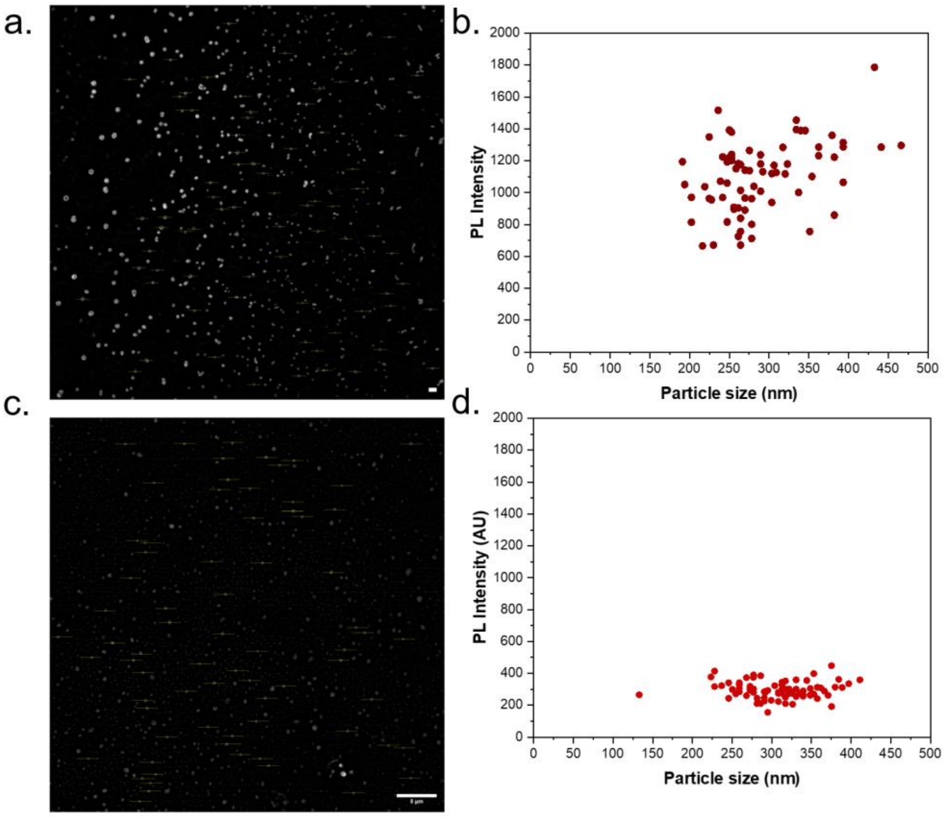

Fig. S16. a) SIM image of the $Cs_4Cd_{0.6}Mn_{0.4}Bi_2Cl_{12}$ nanosheets (scale bar = 1 μm). b) Scatter plot for the intensity vs sizes of the $Cs_4Cd_{0.6}Mn_{0.4}Bi_2Cl_{12}$ nanosheets. 75 particles were chosen for analysis. c) SIM image of the $Cs_4CdBi_2Cl_{12}$ nanosheets (scale bar = 5 μm). d) Scatter plot for the intensity vs sizes of the $Cs_4CdBi_2Cl_{12}$ nanosheets. 75 particles were chosen for analysis.

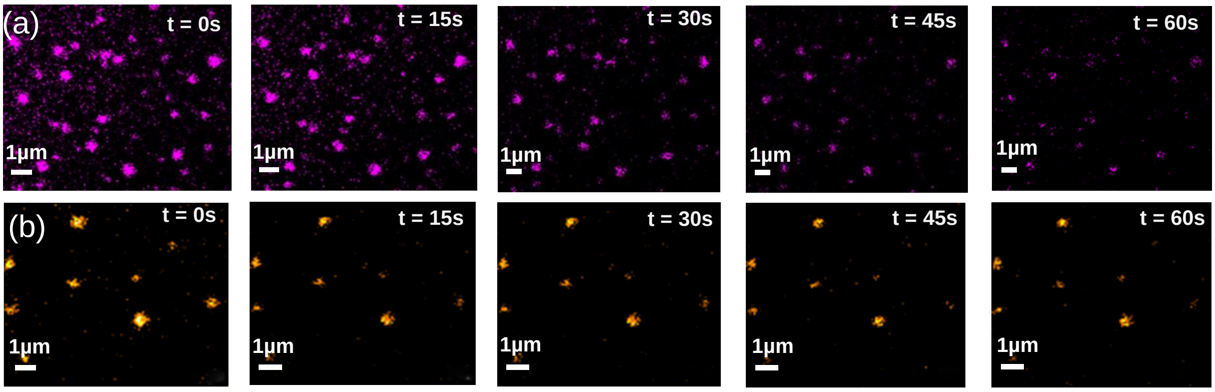

Fig. S17. Temporal snapshots of PL images from nanosheet samples extracted at an interval of 15s each for **(a)** $Cs_4CdBi_2Cl_{12}$, and **(b)** $Cs_4Cd_{0.6}Mn_{0.4}Bi_2Cl_{12}$ up to 60s.

Table S1. ICP-AES analysis for elemental composition in $Cs_4Cd_{0.6}Mn_{0.4}Bi_2Cl_{12}$ nanosheet (NS) sample.

**Elements**	Cs	Bi	Cd	Mn	Cd (%)*	Mn (%)*
**Conc. (mg/L)**	90.68 mg/L	67.73 mg/L	9.62 mg/L	2.47 mg/L	66.70	33.29

*Nominal composition is $Cs_4Cd_{0.6}Mn_{0.4}Bi_2Cl_{12}$, whereas the actual composition from ICP-AES analysis is found to be $Cs_4Cd_{0.67}Mn_{0.33}Bi_2Cl_{12}$.

Table S2. Rietveld refinement parameters of $Cs_4CdBi_2Cl_{12}$ nanosheet (NS) sample.

Space group: R-3m; a = b = 7.611(5) Å, c = 37.24(6) Å, $\alpha = \beta = 90^o$; $\gamma = 120^o$;					
**Atom**	**x**	**y**	**z**	**Occupancy**	U_{iso}(Å^2)
Bi	0.333333	0.666667	0.4123(5)	1	0.0275
Cs	-0.333333	0.333333	0.457(4)	1	0.0488
Cs	0.000	0.000	0.374(4)	1	0.0478
Cd	0.000	0.000	0.500	0.99	0.0247
Cd	0.666667	0.333333	0.333333	0.017	0.0247
Cl	0.15(6)	0.31(12)	0.459(7)	1	0.0540
Cl	0.503(13)	0.497(13)	0.374(7)	1	0.0536

Table S3. Rietveld refinement parameters of $Cs_4Bi_2Cd_{0.67}Mn_{0.33}Cl_{12}$ nanosheet (NS) sample.

Space group: R-3m; a = b = 7.6148(17) Å, c = 37.267(17) Å, $\alpha = \beta = 90^o$; $\gamma = 120^o$;					
**Atom**	**x**	**y**	**z**	**Occupancy**	**U_{iso}(Å^2)**
Bi	0.333333	0.666667	0.4126(3)	1	0.0275
Cs	-0.333333	0.333333	0.4567(21)	1	0.0488
Cs	0.000	0.000	0.3747(19)	1	0.0478
Cd	0.000	0.000	0.5000	0.66	0.0247
Mn	0.000	0.000	0.5000	0.33	0.0247
Cd	0.666667	0.333333	0.333333	0.017	0.0247
Cl	0.156(27)	0.31(5)	0.460(3)	1	0.0540
Cl	0.500(7)	0.500(7)	0.376(4)	1	0.1287

Table S4. Comparative PLQY values along with the thickness of reported perovskite nanosheet (NS) or nanoplate (NPL) samples.

System	Thickness	PLQY	Ref.
2D NSs of $CH_3NH_3PbBr_3$	1.2 nm	32%	ACS Appl. Mater. Interfaces 2020, 12, 6283−6297
	3.2 nm	38%	
	4.8 nm	43%	
	6.4 nm	59%	
	8.4 nm	78%	
2D NSs of $(PEA)_2PbI_4$ (PEA = $C_8H_9NH_3$)	2.0 ± 0.1 nm	34.5%	Angew. Chem. Int. Ed. 2017, 56, 4252 –4255
$(C_4H_9NH_3)_2PbBr_4$ NSs	~1.6 nm (±0.2 nm)	~26%	Science 2015, 349, 1518–1521
$CsPbBr_3$ NSs	2.8−4.2 nm	63%	ACS Nano 2019, 13, 2520−2525
2D $CsPbBr_3$ NSs	~ 3 nm	6-10%	Nanoscale, 2016, 8, 13589-13596
Phase-changed white hexagonal nanosheets (2D octylammonium tin iodide)	1.32 nm	25%	ACS Energy Lett. 2022, 7, 3, 975–983
$(OA)_2SnI_4$ (OA = octylammonium) NSs	~4 nm	~64%	Angew. Chem. Int. Ed. 2023, e202301684
$CdCl_2$-4HP (4-hydroxypyridine) Micro/Nanosheets		63.55%	Angew. Chem. Int. Ed. 2019, 58, 15128-15135
$CdBr_2$-4HP (4-hydroxypyridine) Micro/Nanosheets		25.36%	
$CsPbBr_3$ NPLs	~5.5 nm	0.7%	ACS Nano 2016, 10, 3648-3657
2D NPLs of $CsPbBr_3$	5 monolayer thick	84.4 ± 1.8%	J. Am. Chem. Soc. 2015, 137, 16008−16011
	4 monolayer thick	44.7 ± 2.6%	
	3 monolayer thick	10 ± 0.5%	
$CsPbBr_3$ NPLs	5 monolayers	31%	J. Am. Chem. Soc. 2016, 138, 1010−1016
$L_2[FAPbBr_3]PbBr_4$ (n = 2) NPLs		22%	ACS Nano 2016, 10, 7830−7839
$L_2[MAPbBr_3]PbBr_4$ (n = 2) NPLs		6%	
$Cs_4Cd_{0.6}Mn_{0.4}Bi_2Cl_{12}$	11.35 nm	20.56%	Our work

Table S5. Fitted values of transient PL decay plot by a biexponential decay, relative amplitude of individual components, and the average lifetime of $Cs_4CdBi_2Cl_{12}$ (pristine), and $Cs_4Cd_{0.6}Mn_{0.4}Bi_2Cl_{12}$ (Mn-substituted) nanosheet (NS) samples at 298 K and 77 K.

System	Temp. (K)	Monitoring Wavelength/nm	τ_1/ µs	A_1/%	τ_2/ µs	A_2/%	τ_{avg}/ µs
Pristine	298	600	10.49	49.54	444.9	50.46	435.07
Mn-substituted	298	600	125.3	11.30	482.2	88.70	470.76
Pristine	77	700	12.40	11.09	1103	88.91	1101.47
Pristine	77	760	4.7	54.83	12.95	45.17	10.43
Mn-substituted	77	625	469.2	10.73	1246	89.27	1212.36
